# Comparison of Metabolome and Transcriptome of Flavonoid Biosynthesis in Two Colors of *Coreopsis tinctoria* Nutt.

**DOI:** 10.3389/fpls.2022.810422

**Published:** 2022-03-09

**Authors:** Hong Jiang, Zhiyuan Li, Xiumei Jiang, Yong Qin

**Affiliations:** College of Horticulture, Xinjiang Agricultural University, Xinjiang, China

**Keywords:** *Coreopsis tinctoria* Nutt., metabonomic, transcriptomic, flavonoid biosynthesis, anthocyanins, candidate genes

## Abstract

*Coreopsis tinctoria* Nutt. (*C. tinctoria*) has a long history of application and high economic and medicinal value. Flavonoids, the main active components of *C. tinctoria*, are widely studied in pharmacology and food development. However, the flavonoid biosynthesis pathway in *C. tinctoria* is unclear. In this study, we comprehensively compared the transcriptomes and metabolite profiles of two colors of *C. tinctoria* flowers (LS and JS) at different flowering stages. A total of 165 flavonoids (46 flavonoids, 42 flavonols, 22 anthocyanins, 18 chalcones, 12 dihydroflavonols, nine isoflavones, eight dihydroflavonoids, six flavanols, and two tannins) were identified in LS and JS at different flowering stages. Thirty-three metabolites (11 anthocyanins, 11 flavonols, seven flavonoids, two dihydroflavonols, one dihydroflavone, and one chalcone) were found to be statistically significantly different in the LS vs. JS groups. LS flowers accumulated higher levels of 10 anthocyanins (seven cyanidins and three pelargonidins) than JS flowers. Furthermore, candidate genes related to the regulation of flavonoid and anthocyanin synthesis were identified and included 28 structural genes (especially *F3H*, Cluster-28756.299649, and *3GT*, Cluster-28756.230942) in LS and JS, six key differentially expressed transcription factors (especially *MYB90a*, Cluster-28756.143139) in LS and JS, and 17 other regulators (mainly including transporter proteins and others) in LS. Our results provide valuable information for further studies on the mechanism underlying flavonoid biosynthesis in *C. tinctoria*.

## Introduction

*Coreopsis tinctoria* Nutt. (*C. tinctoria*) is an annual herb in the Compositae family, also referred to as the Asteraceae family. In China, Xinjiang is the main production area of *C. tinctoria*, and the quality of the *C. tinctoria* produced in Keliyang Township, Hetian, is known to be the best (Jing and Lan, 2012). The main active ingredients of *C. tinctoria* are flavonoids, and the medicinal value or effects of this herb, such as its effects in lowering hyperlipidemia, hypertension, and hyperglycemia, its antioxidative effects, its anti-inflammatory properties and its effects in preventing cardiovascular diseases, are also due to the extracted flavonoids (Guo et al., 2014, 2015; Du et al., 2018; Zeng and He, 2019; Shen et al., 2021).

Flavonoids are important products of the secondary metabolism of plants, and they have important ecological significance and physiological functions in regulating flower coloring, physiological activities and adaptation to unfavorable environments (Havsteen, 2002). Subclasses of flavonoids include flavones, isoflavones, flavanols, flavanones, flavonols, and anthocyanins. To date, more than 50 flavonoids, such as quercetin, luteolin, catechin, kaempferol, and rutin, have been extracted and identified from *C. tinctoria* (Guo et al., 2015). Many studies have shown that the total flavonoid content of *C. tinctoria* is correlated with many factors; the total flavonoid contents exhibit statistically significant differences according to habitats (Sun et al., 2012; Chen et al., 2013), harvesting periods (Gu et al., 2015), agronomic measures (Liang et al., 2016) and determination methods (Ma et al., 2019). For example, Du and Wu (2015) found that the total flavonoid content of *C. tinctoria* produced on the Hetian Plateau (132.58 μg/mL) was significantly higher than that of *C. tinctoria* produced on the Dabancheng Plain (99.47 μg/mL), Yang et al. (2016) used the HPLC-DAD (high-performance liquid chromatography with diode-array detection) method to quantitatively analysis of eight compounds in 23 batches, including okanin, and found that the total compound content of *C. tinctoria* produced in Keliyang Township was the highest. Gu et al. (2015) found that there were significant differences in the total flavonoid content in different growth stages of *C. tinctoria*, and the total flavonoid content in the bud stage was the highest, in the range of 12.57–13.73%. Although many basic studies have been performed on the use of the total flavonoid content as an index for evaluating the quality of *C. tinctoria*, a systematic analysis of flavonoid components is still lacking.

At present, the flavonoid synthesis pathway in model plants is relatively well understood. Phenylalanine lyase (PAL), cinnamic acid hydroxylase (C4H), coumadin CoA ligase (4CL), chalcone synthase (CHS), chalcone isomerase (CHI), flavanone 3-hydroxylase (F3H), flavonol synthase (FLS), anthocyanidin synthase (ANS), and other key genes involved in flavonoid synthesis have been isolated and identified from model plants, such as *Arabidopsis thaliana* (Routaboul et al., 2006). With the development and improvement in omics technology, the mechanism underlying flavonoid synthesis in apple (Henry-Kirk et al., 2012), peanut (Wan et al., 2020), Ziziphus jujuba (Zhang et al., 2020) and other plants has been studied. However, plant flavonoid biosynthesis is specific, and flavonoid biosynthesis and related gene expression differ in different plant varieties, developmental stages, tissues and organs (Wen et al., 2020). Transcription factors (TFs) such as *MYB*, *bHLH*, and *WD40*, play important roles in in regulation of flavonoid biosynthesis. For example, in *Arabidopsis thaliana*, the *MYB12* of R2R3-MYB TF can transcribe and regulate the expression of chalcone synthase and flavonol synthase, and its expression level is closely related to flavonol content. Some *MYB* proteins form ternary complexes with the basic helix-loop-helix (BHLH) protein Transparent Testa Glabra 1 (TTG1) WD (beta-transducin)-repeat (WDR) subfamily to regulate the expression of genes related to the biosynthesis of anthocyanins and procyanidins (PA) (Pelletier and Shirley, 1996). The study of a large number of key genes and gene expression regulatory factors in the flavonoid synthesis pathway is an important way to understand this process and increase flavonoid contents. However, the studies on the mechanism of flavonoid synthesis from *C. tinctoria* are unclear.

*Coreopsis tinctoria* is bright in color, with common ligulate flowers exhibiting reddish-brown color at the base and yellow color at the top (the color ratio of reddish-brown to yellow varies from 0 to 9/10) (Edwin and Hampton, 1971; Science Press, 1979). *Coreopsis tinctoria* planted in Xinjiang produces two colors of ligulate flowers, namely, ligulate flowers with red–brown/yellow ratios of 2/3–3/4 and entirely yellow ligulate flowers. In this study, the two colors of *C. tinctoria* are labeled LS (ligulate flowers with red–brown base/yellow top) and JS (entirely yellow ligulate flowers). The accumulation pattern and flavonoid synthesis pathway of LS and JS were studied in different flowering stages, and this study would provide a theoretical basis for identifying and improving the quality and scientific utilization of characteristic resources.

## Materials and Methods

### Plant Materials and Treatments

**FIGURE 1:**
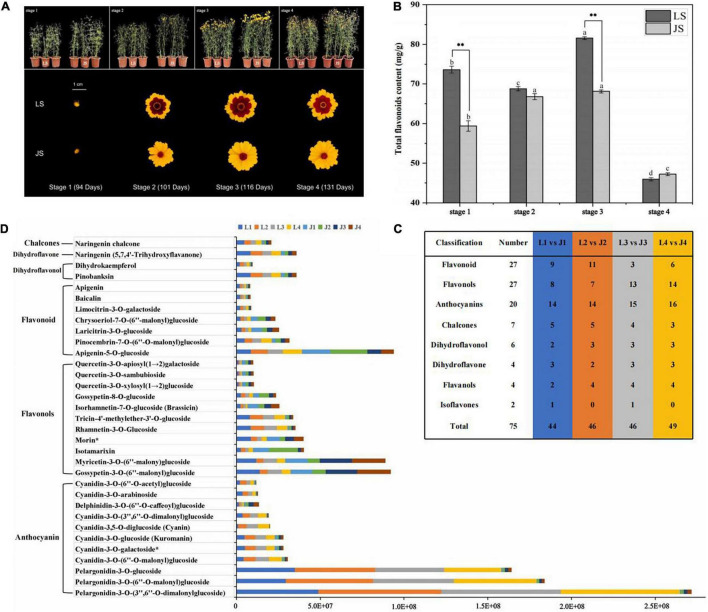
Sampling and analysis of flavonoids metabolites. **(A)** Morphology of four stages of two colors of *C. tinctoria.* **(B)** Total flavonoid content of LS and JS at four stages. **(C)** Statistics on the types of DAFs among different comparison groups. **(D)** The relative content of 33 differential accumulation of flavonoids (DAFs) accumulated at different stages in LS and JS.

The seeds of LS and JS flowers were collected from Keliyang Township, Hetian, China (lat. 37°37′17′′N, long. 78°16′58.80′′E; altitude, 2,196 m), and were potted from March to August 2021 (V peat: V perlite: V vermiculite = 3:1:1) in the solar greenhouse of the Institute of Agricultural Mechanization, Xinjiang Academy of Agricultural Sciences. Eighty pots (pot diameter: 22 cm) were planted for each flower, and each pot contain three plants. Beginning in the seedling stage (30 days after sowing), 1/4–1 standard Hoagland nutrient solution was regularly added (every 7 days; 600 mL per pot) (Supplementary Table 1). The buds and inflorescences of the LS and JS plants were sampled at the bud stage (94 days after sowing, stage 1), the initial flowering stage (101 days after sowing, stage 2), the full flowering stage (116 days after sowing, stage 3) and the final flowering stage (131 days after sowing, stage 4) (Figure 1A). Three biological replicates were collected in each stage of LS and JS plant growth. After collection, the samples were immediately placed into a liquid nitrogen tank at −80°C for storage. The four stages (from stage 1 to stage 4) of LS and JS flowers were labeled as L1, L2, L3, L4 and J1, J2, J3, and J4, respectively.

### Measurement of Total Flavonoid Content

The total flavonoid content was determined by spectrophotometry, with specific reference to the methods of Zhang (2013). Samples without rutin were used as the blank control, and the absorbance of rutin was measured at a wavelength of 510 nm; a standard curve was drawn with concentration *x* as the abscissa and absorbance y as the ordinate. The regression equation obtained was *y* = 23.707*x* − 0.7563 (*R*^2^ = 0.9959). The absorbance was measured at a wavelength of 510 nm in parallel three times, and the total flavonoid content was calculated.

### Qualitative and Quantitative Analysis of Flavonoid Metabolites

The method used to identify flavonoid metabolites was similar to that described by Xiao et al. (2021). First, the freeze-dried *C. tinctoria* buds and inflorescence were ground to a powder with a grinding instrument (MM 400, Retsch) (30 Hz, 1.5 min), and 100 mg of powder was extracted overnight with 1.0 mL of 70% aqueous methanol at 4°C; during this step, the extraction rate was improved by three eddies. After centrifugation for 10 min at 10,000 g, the sample was filtered, and the supernatant was filtered through a microporous membrane (0.22 μm pore size ANPEL, Shanghai, China).^1^ The sample for LC–MS/MS (liquid chromatography with tandem mass spectrometry) analysis was kept in a sample injection vial.

Metabolite data acquisition was conducted with a UPLC (Shim-pack UFLC SHIMADZU CBM30A)^2^ (ultrafast liquid chromatography) instrument coupled to an MS/MS (Applied Biosystems 4500 QTRAP)^3^ instrument. Chromatographic separation was carried out on a Waters ACQUITY UPLC HSS T3 system (Shim-pack UFLC SHIMADZU CBM30A, see text footnote 2), which was equipped with a C18 column (1.8 μm × 2.1 mm × 100 mm), at 40°C. The injection volume was 5 μL at a flow rate of 0.4 mL/min. The organic phase was acetonitrile (with 0.04% acetic acid), and the mobile phase was ultrapure water (with 0.04% acetic acid). The elution gradient was as follows: 0 min, 95:5 water/acetonitrile (v/v); 11.0 min, 5:95 water/acetonitrile; 12.0 min, 5:95 water/acetonitrile; 12.1 min, 95:5 water/acetonitrile; and 15.0 min, 95:5 water acetonitrile. The metabolites in the samples were analyzed both qualitatively and quantitatively by using MS data, Analyst 1.6.3 software, and metabolite information from the public MetWare database (MWDB, Wuhan MetWare, China).

Based on the results of principal component analysis (PCA), the metabolites with | log2 Fold Change (FC)| ≥ 1 and variable importance in project (VIP) value ≥ 1 in the OPLS-DA (orthogonal projections to latent structures-discriminant analysis) model were considered to be statistically significant different among samples (Saccenti et al., 2013).

### RNA Extraction, Library Establishment, and Sequencing

The total RNA was extracted from 24 samples of *C. tinctoria* by the TRIzol (Invitrogen) method, and the quality of the RNA samples was assessed by agarose gel electrophoresis with a Qubit 2.0 Fluorometer (Life Technologies, CA, United States) and an Agilent 2100 Bioanalyzer (Agilent Technologies, Palo Alto, CA, United States) to ensure that qualified samples were used for transcriptome sequencing. The cDNA library was established by a TruSeq™ RNA sample preparation kit (Illumina, San Diego, CA, United States). mRNA with a polyA tail was enriched with oligo (dT) (deoxythymine) magnetic beads, and the mRNA molecules were broken into short fragments with the addition of fragmentation buffer. Using the short fragment mRNA as the template, first-strand cDNA was synthesized by six-base random primers (random hexamers). Buffer, dNTPs (deoxynucleotide triphosphates; dUTP (2′-deoxyuridine, 5′-triphosphate), dATP (deoxyadenosine triphosphate), dGTP (deoxyguanosine triphosphate), and dCTP (deoxycytidine triphosphate) and DNA polymerase I were then added to synthesize double-stranded cDNA.

The double-stranded cDNA was then purified by AMPure XP beads. The double-stranded cDNA structure had a sticky end that was filled until blunt via the addition of End Repair Mix, and an A base was added to the 30 end to overlap with a T base overhang. The fragment size was then selected by AMPure XP beads, and the final cDNA library was enriched by PCR (polymerase chain reaction) as described in the instructions. After cDNA enrichment by PCR, a Qubit 2.0 instrument was used for preliminary quantification, and an Agilent 2100 instrument was used to detect the insert size of the library. The effective concentration of the library was subsequently determined by Q-PCR (quantitative polymerase chain reaction) (effective concentration of the library > 2 nM), and the Illumina HiSeq platform was used for sequencing (Wuhan Metware).

### *De novo* Assembly and Functional Annotation

The raw data of 24 samples were obtained by *de novo* transcriptome sequencing, and the raw data underwent filtering and quality control before reassembly. To obtain clean reads, an eliminated adapter was used to remove the adapter reads, reads containing more than 10% unknown nucleotides and low-quality reads (reads containing more than 50% bases with Q ≤ 20 reads). There is no reference genome for *C. tinctoria*, and the clean reads were spliced by Trinity^4^ to obtain the transcript sequence, which was used as a reference sequence for subsequent analyses. Hierarchical clustering of transcripts was performed by comparing the read number and expression pattern of transcripts using Corset.^5^ Hierarchical clustering was conducted with Corset to ultimately obtain the longest cluster sequence as a follow-up analysis on unigenes.

Then, we used BLAST software to compare the unigene sequences with seven public databases, including the NCBI non-redundant protein (NR),^6^ TrEMBL (a computer annotated supplement to SwissProt), Pfam,^7^ SwissProt,^8^ Kyoto Encyclopedia of Genes and Genomes (KEGG),^9^ Gene Ontology (GO)^10^ and euKaryotic Ortholog Groups (KOG)^11^ databases. The RNA-Seq data set was uploaded to NCBI^12^ (accession no. PRJNA698724).

### Quantitative Gene Expression and Differentially Expressed Genes Analysis

The clean reads of 24 samples were mapped back to a reference sequence using Bowtie 2,^13^ and the fragments per kilobase of transcript per million fragments mapped (FPKM) method was used to normalize the expression of the mapped reads. The read count of genes was determined using feature counts. DESeq2 was used to analyze the difference in the read count data. The multiple hypothesis test correction of the *P* value (probability) was carried out using the Benjamini–Hochberg method, and the false discovery rate (FDR) was determined. Genes that satisfied the conditions of | log_2_ FC| ≥ 1 and FDR < 0.05 were considered to be differentially expressed (Love et al., 2014).

### Annotation and Analysis of Transcription Factors

Transcription factor prediction was performed using iTAK software, which integrates the PlnTFDB and PlantTFDB databases and uses the well-defined TF family and rules in the database. TFs were identified through hmmscan comparison, TFs related to flavonoid synthesis were identified through a reference review. The selected TFs with DAFs/DAAs Spearson’s correlation (ρ) values > 0.80 or <−0.80 were selected to draw the interaction network diagram.

### Weighted Gene Co-expression Network Analysis

The overlapping differentially expressed genes (DEGs) (2,140 out of 7,483 DEGs) and differential anthocyanins accumulation (DAAs) were selected for co-expression network analysis via weighted gene co-expression network analysis (WGCNA). The content of overlapping DAAs in LS was used as a trait for WGCNA, and modules were obtained through WGCNA with default settings. Furthermore, the correlation coefficients between the hub genes in the module were determined. The hub genes with DAAs Pearson’s correlation coefficient (| PCC| > 0.80) values were selected to draw the interaction network diagram.

### Validation of RNA-Seq Data by Quantitative Real Time-PCR

The nine DEGs related to flavonoid biosynthesis identified in the RNA-seq data were selected for validation by qRT–PCR (real-time quantitative reverse transcription). The total RNA of three biological repeat sequences was extracted from flower buds and inflorescences by TRIzol reagent (Invitrogen, Shanghai, China) for qRT–PCR analysis. The RNA of each sample was reverse transcribed into cDNA by the PrimeScript fragments RT reagent Kit with gDNA Eraser (Perfect Real Time). A Bio-Rad CFX Connect fluorescence quantitative PCR detection system was used. The amplification cycle procedure was as follows: 95°C for 3 min, 95°C for 10 s, 55°C for 20 s, 72°C for 20 s, 75°C for 5 s, repeated for 40 cycles. Primers targeting the reference gene *GAPDH* were designed according to the conserved region in a *GAPDH* gene sequence alignment of *Helianthus annuus*. The 2^–ΔΔ*CT*^ method was used to calculate relative gene expression, and the error bars represent the mean ± standard deviation (SD). The primer information is shown in the attached Supplementary Table 2.

### Data Analysis

Excel 2010 was used for data processing, SPSS Statistics 23.0 software was used for analysis of variance, and Origin 2018 software was used for generating the figures. The R software (R version 3.6.1) stats package was used to drawing the correlation network diagram.

## Results

### Metabolic Differences Between the Two Colors of *Coreopsis tinctoria*

To explore the mechanism underlying flavonoid metabolism in *C. tinctoria*, the total flavonoid contents of LS and JS were measured. Among the four flowering stages of LS, the total flavonoid content was significantly higher in stage 3 (81.60 mg/g) than in the other stages. This was followed by the flavonoid contents of LS in stage 1, stage 2, and stage 4, and the total flavonoid contents during these stages were 73.65, 68.78, and 45.98 mg/g, respectively. The total flavonoid content of JS first increased and then decreased with of the progression of flowering stage. The total flavonoid content of JS was highest at stage 3, 68.11 mg/g. This was followed by the contents of JS in stage 1, stage 2, and stage 4, and the total flavonoid contents during these stages were 59.38, 66.79, and 47.24 mg/g, respectively. The differences in the total flavonoid contents of LS in stage 1 and stage 3 from those of JS in the same stages were statistically significant, i.e., the total flavonoid contents in LS were 24 and 20% higher than those in JS, respectively. The results showed the dynamic difference in total flavonoid content of LS and JS in the four different stages, especially in stage 1 and stage 3 (Figure 1B).

To further analyze the differences in flavonoid metabolites in *C. tinctoria* (LS and JS) at different stages, the flavonoid metabolites were detected by UHPLC–ESI–MS/MS. The total ion flow map (TIC) obtained from QC samples indicates the sum of all ion strengths at different time points (Supplementary Figure 1A). In the detection multipeak map (XIC), the chromatographic peaks of different colors correspond to different flavonoid metabolites (Supplementary Figure 1B). The superposition of the total ion flow map (TIC map) of the QC samples shows that the response intensity and retention time of each peak strongly overlapped, indicating that the data were reliable (Supplementary Figure 1C). A total of 165 flavonoids were identified in the buds and inflorescences of LS and JS at different flowering stages (Supplementary Table 3), and these flavonoids included 46 flavonoids, 42 flavonols, 22 anthocyanins, 18 chalcones, 12 dihydroflavonols, nine isoflavones, eight dihydroflavonoids, six flavanols, and two tannins (Supplementary Figure 2). The relative contents of flavonoids were analyzed by hierarchical cluster thermography (HCA), and there were significant differences in the patterns of flavonoid accumulation in LS and JS at different stages. The more relative contents of flavonoids accumulated in LS and JS in stage 1, and more flavonoids accumulated during the flowering stages of LS than at that of JS (Supplementary Figure 3). Principal component analysis (PCA) showed that the L1 vs. J1, L2 vs. J2, L3 vs. J3, and L4 vs. J4 groups were clearly separated, and principal components PC1 (38.15%) and PC2 (23.00%) showed a degree of change of 61.50% (Supplementary Figure 4), indicating the difference between the LS and JS groups. The PCA results were consistent with the HCA results, which showed the repeatability and reliability of the data.

Based on the data obtained from the metabolome, 44, 46, 46, and 49 DAFs were identified between the L1 vs. J1, L2 vs. J2, L3 vs. J3, and L4 vs. J4 groups, respectively (Figure 1C). In addition, 33 DAFs (E05 and above and | log_2_ FC| > 1) were identified in LS and JS at different stages. Compared to JS, there were 20 DAFs (10 anthocyanins, four flavonoids, two flavonols, two dihydroflavonols, one dihydroflavone, and one chalcone) that significantly accumulated in LS at one or more stages. Compared to LS, 13 DAFs (nine flavonols, three flavonoids, and one anthocyanin) accumulated in JS at one or more stages. Seventeen DAFs (10 anthocyanins, two flavonols, two dihydroflavonols, one flavonoid, one dihydroflavone, and one chalcone) significantly accumulated in LS and JS during the four stages (Figure 1D).

The DAFs with the largest fold changes among the different stages were mainly enriched in anthocyanins in LS. Pelargonidin-3-O-(3′′,6′′-*O*-dimalonylglucoside) (log_2_FC = −6.25 to −4.82), pelargonidin-3-*O*-(6′′-*O*-malonyl) glucoside (log_2_FC = −5.96 to −4.86), pelargonidin-3-*O*-glucoside (log_2_FC = −5.61 to −4.09), cyanidin-3,5-*O*-diglucoside (Cyanin) (log_2_FC = −5.49 to −3.45), cyanidin-3-*O*-(3′′,6′′-*O*-dimalonyl) glucoside (log_2_FC = −3.71 to −3.16), cyanidin-3-*O*-(6′′-*O*-acetyl)glucoside (log_2_FC = −3.65 to −1.56), cyanidin-3-*O*-arabinoside (log_2_ FC = −4.11 to −2.38), and cyanidin-3-*O*-(6′′-*O*-malonyl)glucoside (log_2_FC = −3.11 to −2.31) substantially accumulated in LS during four stages. In JS, flavonols obviously accumulated in a few stages, among which quercetin-3-*O*-xylosyl(1→2)glucoside, quercetin-3-*O*-apiosyl(1→2)galactoside and quercetin-3-*O*-sambubioside accumulated in stage 3, and the Log_2_FC values were 1.45, 1.45, and 1.41, respectively. In addition, delphinidin-3-*O*-(6-*O*-caffeoyl) glucoside accumulated in JS in stage 3 and stage 4, changing by a multiple of 1.42–1.72 (log_2_FC value) (Supplementary Table 4). These DAFs might be the key metabolites that influence the changes in flavonoids in LS and JS. And these significantly accumulated anthocyanins might affect the coloration of LS and JS.

### Transcriptome Sequencing, Assembly, and Statistics

To further study the difference in the mechanism underlying flavonoid synthesis between LS and JS, *de novo* transcriptome sequencing was performed on 24 samples of buds and inflorescences of *C. tinctoria*. For each sample, 42,768,396–53,410,008 raw reads were obtained by high-throughput sequencing, and after splices and low-quality sequences were excluded, 40,703,558–51,523,378 clean reads were obtained. Q30 bases accounted for greater than 91.79% of the total, and the average GC content was 43.90% (Supplementary Table 5). After assembly with Trinity software, 463,574 transcripts with an average length of 786, 447,813 unigenes with an average length of 804 bp, N50 lengths of 1,049 and 1,061 bp, and 23,017 transcripts and unigenes with sequence lengths greater than or equal to 2,000 bp were identified (Supplementary Table 6). Comparing a total of 447,813 unigenes with information from seven public databases (NR, Trembl, Swiss-Prot, Pfam, KOG, GO, and KEGG), it was found that the comparison rate of all unigenes in at least one database was as high as 60.40% (Supplementary Table 7). In summary, the RNA-seq data were reliable and had high quality, and these data could be used for further analysis.

### Differentially Expressed Genes Analysis of Expression and KEGG Enrichment

**FIGURE 2:**
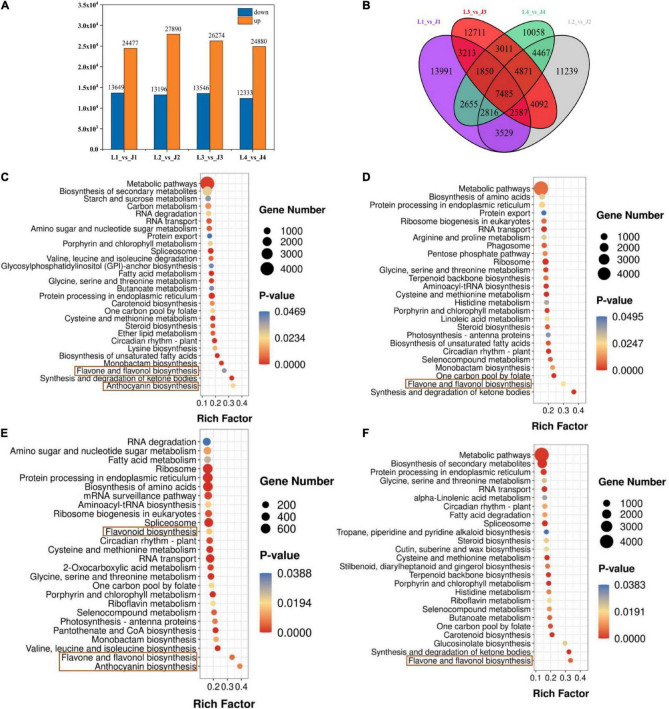
Differentially expressed genes (DEGs) in LS and JS. **(A)** Statistics of DEGs in different comparison groups of LS and JS. Red indicates upregulated DEGs. Green indicates downregulated DEGs. **(B)** Venn diagram of DEGs. **(C–F)** DEGs enrichment KEGG scatter plot in four stages of LS and JS comparison groups. **(C)** L1 vs. J1 group. **(D)** L2 vs. J2 group. **(E)** L3 vs. J3 group. **(F)** L4 vs. J4 group.

First, Pearson correlation analysis was carried out on 24 samples, and the results showed that the repeatability of the same group of samples was good, and there were significant differences in correlation between different groups (Supplementary Figure 5). The DEGs were analyzed by DESeq2 software, and the selected DEGs (| log_2_FC| ≥ 1 and FDR < 0.05) were then compared and analyzed. There were 38,126, 41,086, 39,820, and 37,213 DEGs in the L1 vs. J1, L2 vs. J2, L3 vs. J3, and L4 vs. J4 groups, respectively, of which 24,477, 27,890, 26,274, and 24,880 were upregulated and 13,649, 13,196, 13,546, and 12,333 were downregulated, respectively. Compared to those expressed by LS, the number of upregulated genes expressed by JS in stage 2 and stage 3 was the highest, and the number of downregulated genes expressed by JS in stage 1 and stage 3 was the highest. Stage 3 was the critical period of LS and JS (Figure 2A). The Venn diagram results showed that 7,458 DEGs were co-expressed among the four comparison groups, which indicated that these DEGs might perform key functions in flavonoid regulation in LS and JS (Figure 2B).

The DEGs identified in the LS vs. JS comparison groups were involved in a total of 137 KEGG metabolic pathways, and 28, 26, 26, and 25 significantly enriched metabolic pathways were identified in the L1 vs. J1, L2 vs. J2, L3 vs. J3, and L4 vs. J4 groups, respectively (Supplementary Table 8). Among them, flavone and flavonol biosynthesis (ko00944) was significantly enriched among the four comparative groups. Anthocyanin biosynthesis (ko00942) was significantly enriched in the L1 vs. J1 and L3 vs. J3 comparison groups, and flavonoid biosynthesis (ko00941) was significantly enriched in the L3 vs. J3 comparison groups. It is suggested that flavonoid biosynthesis-related pathways play an important role in different stages of LS and JS development (Figures 2C–F).

### Analysis of Differentially Expressed Genes Related to Flavonoid and Anthocyanin Biosynthesis

**FIGURE 3:**
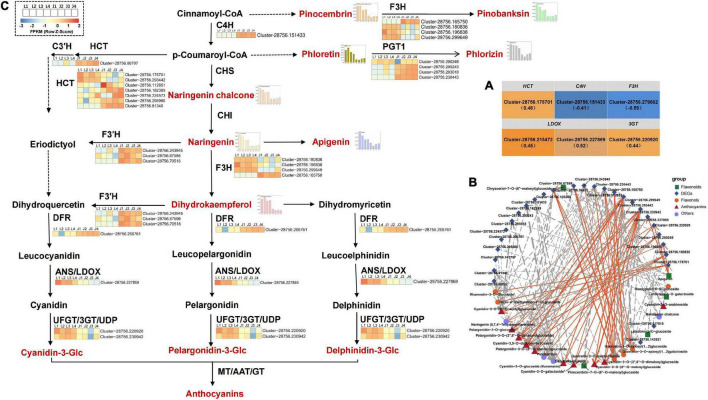
DEGs analysis of flavonoid and anthocyanin synthesis in *C. tinctoria.* **(A)** Six DEGs correlated with total flavonoids content. The brackets indicated the correlation coefficient ρ. **(B)** There was a significant correlation between 28 DEGs and 25 DAFs (*P* < 0.01, —ρ— > 0.80). The solid line indicates the positive correlation, the dotted line indicates the negative correlation, and the thickness of the line indicates the correlation strength. **(C)** The 28 DEGs (in **B**) involved in flavonoid and anthocyanin biosynthesis pathway of LS and JS. *C*3′*H*, coumaroylquinate 3′-monooxygenase; *C4H*, trans-cinnamate 4-monooxygenase; *HCT*, shikimate O-hydroxycinnamoyltransferase; *PGT1*, phlorizin synthase; *F3H*, naringenin 3-dioxygenase; *F*3′*H*, flavonoid 3′-monooxygenase; *DFR*, bifunctional dihydroflavonol 4-reductase; *LDOX*, leucoanthocyanidin dioxygenase; *3GT*, anthocyanidin 3-O-glucosyltransferase.

Based on KEGG enrichment analysis and gene functional annotation, 61 DEGs (at least one stage FPKM > 10) were shown to be significantly enriched in pathways related to flavonoid biosynthesis. The 61 DEGs included 29 DEGs (19 *HCT* genes, eight *PGT1* genes, one *C3′H* gene, one *CCoAOMT* gene) involved upstream of flavonoid biosynthesis and 32 DEGs (two *C4H* genes, two *CHS* genes, five *CHI* genes, six *F3H* genes, nine *F*3′*H* genes, three *DFR* genes, two *3GT* genes, two *LDOX* genes, and one *UF3RT* gene) involved in flavonoid biosynthesis and anthocyanin biosynthesis (Supplementary Table 9). Furthermore, the Spearman correlation between the expression level of 61 DEGs and the total flavonoid content and the relative content of 33 DAFs were analyzed. The results showed that there was a positive correlation between the expression of four DEGs (one *HCT* gene, Cluster-28756.178701; one *3GT* gene, Cluster-28756.220920; two *LDOX* genes, Cluster-28756.215472, Cluster-28756.227869) and total flavonoid content, whereas the expression of two DEGs (one *C4H* gene Cluster-28756.151433; one *F3H* gene, Cluster-28756.279662) had a negative correlation with total flavonoid content (*P* < 0.05; Figure 3A). In addition, the expression levels of 28 genes (seven *HCT* genes, four *PGT1* genes, one *C*3′*H* gene, one *C*4*H* gene, four F3H genes, five *F*3′*H* genes, three *DFR* genes, one *LDOX* gene, and two *3GT* genes) were significantly associated with the levels of 25 DAFs (10 anthocyanins; six flavonols; five flavonoids; two dihydroflavonol; one chalcones; and one dihydroflavone). The expression levels of the *F3H* gene (Cluster-28756.299649) and *3GT* gene (Cluster-28756.230942) were significantly positively correlated with the levels of 7 types of anthocyanins (pelargonidin-3-*O*-glucoside, cyanidin-3-*O*-glucoside (Kuromanin), cyanidin-3-*O*-galactoside, pelargonidin-3-*O*-(6′′-*O*-malonyl)glucoside, cyanidin-3-*O*-(6′′-*O*-malonyl)glucoside, pelargonidin-3-*O*-(3′′,6′′-*O*-dimalonylglucoside), cyanidin-3-*O*-(3′′,6′′-*O*-dimalonyl)glucoside). On the other hand, the expression of these two DEGs were significantly negatively correlated with the levels of three types of flavonols [quercetin-3-*O*-apiosyl(1→2)galactoside, quercetin-3-*O*-sambubioside, quercetin-3-O-xylosyl(1→2)glucoside]. The expression levels of these two DEGs in LS in the four stages were significantly higher than those in JS, especially in stage 3, suggesting that the two DEGs may play an essential role in anthocyanin accumulation in LS. Otherwise, the expression of *HCT* gene (Cluster-28756.81340), *PGT1* gene (Cluster-28756.230443), and *F3H* gene (Cluster-28756.165750) were significantly negatively correlated with the level of naringenin chalcone. The expression of the *PGT1* gene (Cluster-28756.230443) and *F3H* gene (Cluster-28756.165750) were significantly negatively correlated with the levels of three anthocyanins [cyanidin-3-*O*-arabinoside, pelargonidin-3-*O*-(6′′-*O*-malonyl)glucoside, and pelargonidin-3-*O*-(3′′,6′′-*O*-dimalonylglucoside)] and 1 flavonoid [pinocembrin-7-*O*-(6′′-*O*-malonyl)glucoside]. The expression levels of the *HCT* gene (Cluster-28756.81340) and the *PGT1* gene (Cluster-28756.230443) were significantly negatively correlated with the levels of two flavonols (rhamnetin-3-*O*-glucoside and tricin-4′-methyl ether-3′-*O*-glucoside) and one dihydroflavonol (dihydrokaempferol). These three genes were only highly expressed in JS in the four stages but not in LS in the four stages, indicating that the three DEGs might negatively regulate flavonoid synthesis in JS (Figures 3B,C).

### Quantitative Real Time-PCR

To verify the accuracy of the RNA-seq data, nine DEGs involved in the flavonoid metabolic pathway were selected, and their expression was verified by qRT–PCR. The results showed that the trends of qRT–PCR and transcriptome expression were basically consistent with those of gene expression, indicating that the measured transcriptome data were effective and reliable (Supplementary Figure 6).

### Analysis of Transcription Factor-Mediated Regulation of Flavonoid and Anthocyanin Biosynthesis

The flavonoid biosynthesis pathway is mainly regulated by external factors and TFs, and the TFs involved in the regulation include *MYB, bHLH* (*MYC*), and WD40 family (Zhang et al., 2014). In this study, many kinds of TFs were identified in LS and JS in the four stages. Among them, the largest number of TF families (the top 10 categories) was identified in the L1 vs. J1, L2 vs. J2, L3 vs. J3, and L4 vs. J4 groups, and these TF families included *MYB*, *C3H*, *C2H2*, *NAC*, *AP2/ERF-ERF*, *GRAS*, *bHLH*, *bZIP*, and so on. The number of MYB (including MYB-related) gene families was the highest among the four comparison groups (Supplementary Figure 7). The eight *MYB* and five *bHLH* TFs (at least one stage FPKM > 10) related to flavonoid biosynthesis were screened. The expression levels of these TFs were different in LS and JS in one or more stages (Niu et al., 2016; Supplementary Figure 8). There was a significant correlation between the expression of six TFs and the levels of six cyanidins. The expression levels of the *MYB90a*, *MYB90b*, and *MYB111c* TFs all had significant positive correlations with the levels of one or more cyanidins. However, the expression levels of *MYB111a*, *MYC2b*, and *MYC2c* all had significant negative correlations with the levels of one or more cyanidins. Among them, the expression of *MYB90a* (Cluster-28756.143139) was significantly positively correlated with the levels of five types of cyanidins [cyanidin-3-*O*-glucoside (kuromanin), cyanidin-3-*O*-galactoside, cyanidin-3-*O*-(6′′-*O*-acetyl)glucoside, cyanidin-3,5-*O*-diglucoside (cyanin), and cyanidin-3-*O*-(3′′,6′′-*O*-dimalonyl)glucosidec], while there was a significant negative correlation between the expression of *MYC2b* and the levels of these five types of cyanidins (Supplementary Figure 9). *MYB90a* was only highly expressed in LS in the four stages, indicating that the *MYB90a* TF might positively regulate anthocyanin biosynthesis (cyanidins) in LS. In contrast, the expression of *MYC2b* was only observed in JS during the four stages, indicating that the *MYC2b* TF might negatively regulate anthocyanin biosynthesis (cyanidins) in JS.

### Identification of Anthocyanin Synthesis-Related Differentially Expressed Genes in LS by Weighted Gene Co-expression Network Analysis

**FIGURE 4:**
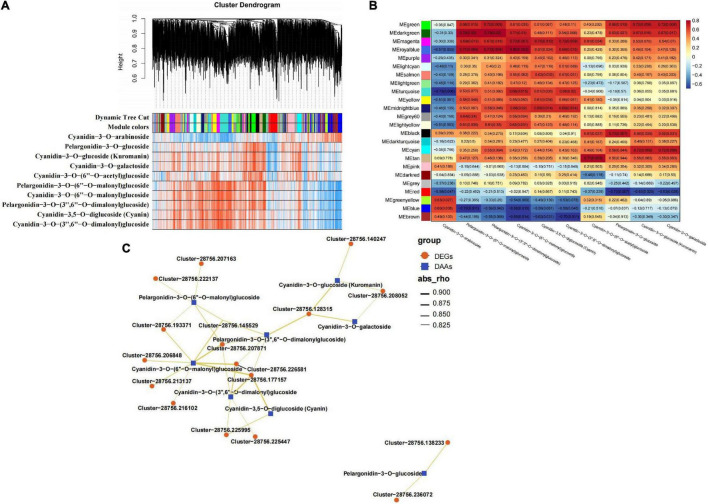
WGCNA results of 2140 DEGs (with 10 DAAs —PCC— > 0.80). **(A)** Hierarchical clustering tree (cluster dendrogram) results showed 24 expression modules, labeled with different colors. **(B)** Module-anthocyanin relationship analysis. The value inside each box represents Pearson’s correlation coefficient between the module with anthocyanin, and the number in each parentheses represents *p*-value. The color scale on the right represents the degree of correlation between modules and anthocyanins and the red represent high correlation. **(C)** The interaction network diagram between DEGs and 10 DAAs. Red triangles represent DEGs and blue triangles represent DAAs.

To investigate the gene regulatory network of anthocyanin accumulation in LS, WGCNA was conducted using the FPKM values of the overlapping DEGs (2,140 out of 7,483 DEGs, FPKM > 1 at least in one stage in LS) and DAFs (10 accumulated anthocyanins in LS) as the source data. A total of 24 DEG modules were identified in the cluster dendrogram (Figure 4A), four of which MEgreen, MEdarkgreen, MEmagenta, and MEroyablue were highly correlated with the contents of more types of anthocyanins (Figure 4B). To explore the expression patterns of the DEGs in these modules, heatmaps were generated using the FPKM values of the DEGs in the four modules. The heatmap results of the four modules showed that most DEGs were expressed at higher levels in the flowering stages (stages 2–4) (Supplementary Figure 10). Moreover, the interaction network diagram was constructed between the hub genes (top 20 DEGs with WGCNA weights of each module) in the MEgreen (weight > 3.44), MEdarkgreen (weight > 0.65), MEmagenta (weight > 3.29), and MEroyablue (weight > 2.04) modules and the anthocyanin content. In the modules, 17 DEGs were selected to construct the regulatory network, including five enzyme genes, eight protein-related genes, two flower-related genes, and two genes with unknown functions (Supplementary Table 10). These genes both positively regulated one or more types of DAAs (cyanidin-3,5-*O*-diglucoside (cyanin), cyanidin-3-*O*-(3′′,6′′-*O*-dimalonyl)glucoside, cyanidin-3-*O*-(6′′-*O*-malonyl)glucoside, cyanidin-3-*O*-galactoside, cyanidin-3-*O-*glucoside (Kuromanin), pelargonidin-3-*O*-(3′′,6′′-*O*-dimalonyl)glucoside, pelargonidin-3-*O*-(6′′-*O*-malonyl)glucoside, and pelargonidin-3-*O*-glucosidec). As shown in Figure 4C, cyanidin-3-*O*-(6′′-*O*-malonyl)glucoside was at the core of the network diagram, and it was positively regulated by eight genes. Then, pelargonidin-3-*O*-(6′′-*O*-malonyl)glucoside, pelargonidin-3-*O*-(3′′,6′′*-O-*dimalonyl)glucoside, and cyanidin-3-*O*-(3′′,6′′-*O*-dimalonyl)glucoside were positively regulated by five genes. This finding indicated the main contribution of these four DAAs to LS coloration, and the important regulatory role of these 17 genes in LS anthocyanin synthesis was hypothesized.

## Discussion

### Identification of Flavonoid Metabolites in *Coreopsis tinctoria*

Flavonoids contribute in important ways to the medicinal effects of *C. tinctoria*, and studies on the identification of flavonoids in *C. tinctoria* are mostly focused on LS (Wang et al., 2015; Shen et al., 2021). However, there are few studies on JS, an important flower color type of *C. tinctoria*. In this study, the total flavonoid content in LS and JS during four flowering stages was determined. The results showed that there were significant changes in the total flavonoid content in the same or different stages of the two colors of *C. tinctoria* (LS and JS). In both LS and JS, the total flavonoid content was highest in stage 3, 81.6 and 68.11 mg/g, respectively. Then, 165 flavonoid metabolites in LS and JS during the four stages were identified by LC–MS/MS; these metabolites included 46 flavones, 42 flavonols, 22 anthocyanins, 18 chalcones, 12 dihydroflavonols, and nine isoflavones. The results showed that flavones, flavonols and anthocyanins were the main types of flavonoids in the flowering stages of *C. tinctoria*. Thirty-three DAFs that exhibited statistically meaningful changes were identified in the LS vs. JS groups, and these DAFs included 11 anthocyanins, 11 flavonols, seven flavonoids, two dihydroflavonols, one chalcone, and one dihydroflavone. There were significant differences in the patterns of accumulation of these DAFs in LS and JS during different stages. The degree of anthocyanin accumulation in LS was higher than that in JS; otherwise, the degree of flavonoid accumulation in JS was higher than that in LS. Compared with the limitations of previous studies that determined the types of flavonoids in *C. tinctoria*, the results of this study greatly enrich our knowledge of the types and components of flavonoids in *C. tinctoria*.

### Anthocyanin Differences in *Coreopsis tinctoria*

Anthocyanin is the main chromogenic substance in flavonoids, and cyanidin, pelargonidin and delphinidin are common anthocyanins (Wu et al., 2018) in plants. Usually, cyanidin has a purplish red color, delphinidin has a blue–purple color, and pelargonidin has a brick-red color (Yu et al., 2019). Early on, Shimokoriyama indicated that the red–brown component of the ligulate flower of LS was cyanidin-3-*O*-glucoside (Shimokoriyama, 1957). In this study, we screened 10 anthocyanins that exhibited significantly different accumulation patterns in LS at the four stages, including seven types of cyanidins [cyanidin-3-*O*-(6′′-*O*-acetyl)glucoside, cyanidin-3-*O*-arabinoside, cyanidin-3-*O*-glucoside (kuromanin), cyanidin- 3-*O*-galactoside, cyanidin-3-*O*-(3′′,6′′-*O*-dimalonyl)glucoside, cyanidin-3-*O*-(6′′-*O*-malonyl)glucoside, and cyanidin-3,5-*O*-diglucoside (cyanin)] and three types of pelargonidins [pelargonidin-3-*O*-(3′′,6′′-*O*-dimalonylglucoside), pelargon idin-3-*O*-(6′′-*O*-malonyl) glucoside, and pelargonidin-3-*O*-glucoside]. The composition and content of anthocyanins greatly affect flower color. Jiang et al. (2020) and Wang F. et al. (2021) observed anthocyanins, such as cyanidin-3-*O*-glucoside and cyanidin-3,5-*O*-diglucoside (cyanin), from purple *Salvia miltiorrhiza* and purple *Ficus carica* Linn., respectively. Fu et al. (2021) found that cyanidin-3-*O*-(6′′-*O*-malonyl)glucoside was the main contributor to the color of *Camellia japonica*. The results of these studies were similar to those of this study, and these DAAs may be the main reason for the difference in flower color between LS and JS.

### Key Structural Genes Responsible for Flavonoid Synthesis in *Coreopsis tinctoria*

At present, the flavonoid synthesis pathway has been clearly studied in *Arabidopsis thaliana* and other model plants (Kleindt et al., 2010; Martens et al., 2010). The biosynthesis pathway of flavonoids in most plants is relatively conserved, but the transcription levels of structural genes (*PAL*, *4CL*, *C4H*, *CHS*, *CHI*, *F3H*, etc.) involved in the flavonoid biosynthesis pathway are different in different plants or in the same plants in different developmental stages and tissues, thus affecting the regulation of flavonoid synthesis (Chen et al., 2021). The anthocyanin synthesis pathway is a branch of the flavonoid biosynthesis pathway, and related studies have focused on different plants with color formation, such as sweet potato (He et al., 2020), radish (Park et al., 2011), and passion fruit (Qiu et al., 2020). In this study, *C4H* (1), *F3H* (4), *F3*′*H* (5), *DFR* (3), *LDOX* (1), and *3GT* (2) structural genes that were directly involved in the flavonoid biosynthesis pathway and anthocyanin biosynthesis pathway were identified in *C. tinctoria* (LS and JS), which was consistent with the results of related studies on Rhododendron (Nakatsuka et al., 2008) and Chinese herbaceous peony (Zhao et al., 2012). Considering the difference in anthocyanin contents between LS and JS in different stages, two genes (the *F3H* gene, Cluster-28756.299649, and the *3GT* gene, Cluster-28756.230942) whose expression was significantly positively correlated with the levels of more types of anthocyanins were identified. *F3H* is the key enzyme in the flavonoid metabolic pathway, and it can catalyze naringin to produce dihydroflavonol (Castellarin et al., 2007). Dihydroflavonol is the starting substrate of anthocyanin synthesis, and it produces anthocyanins after catalysis by a series of key enzymes. Finally, colored anthocyanins are catalyzed by anthocyanidin 3-*O*-glucosyltransferases (*UFGT/3GT*) to combine with glucoside to produce colored anthocyanins. These two DEGs were specifically expressed by LS, especially in stage 3, which was similar to the results obtained in relevant studies of grapes by Mohammad et al. (2011). These two genes promoted the accumulation of anthocyanins in LS. We hypothesize that these two genes may play key roles in anthocyanin accumulation in *C. tinctoria*. In addition, the expression levels of seven number of *HCT* genes upstream of flavonoid synthesis were significantly different in LS and JS, which is similar to the screening results of Wang R. et al. (2021) in safflower. Correlation analysis with DAFs showed that the expression levels of three number of *HCT* genes (Cluster-28756.81340, Cluster-28756.178701, and Cluster-28756.112851) were statistically significantly correlated with the levels of a variety of flavones, flavonols, and anthocyanins, indicating that the *HCT* gene promotes the accumulation of flavonoids.

### Transcription Factors and Other Candidate Genes Related to Anthocyanin Synthesis

Plant flavonoid biosynthesis is affected not only by the expression levels of structural genes but also by the levels of MYB, bHLH, and WD40 TFs and their MYB–bHLH–WD40 (MBW) complex (Hichri et al., 2011; Liu et al., 2021). This study found that the number of MYB TFs (including MYB-related TFs) in *C. tinctoria* in different stages was the highest, and some bHLH and bZIP TFs were also identified, but no WD40 TFs were identified, indicating that MYB was the main TF category that regulates the synthesis of flavonoids in *C. tinctoria.* MYB TFs can be divided into 1R-MYB (1R-MYB and MYB-related), R2R3-MYB, 3R-MYB, and 4R-MYB (Niu et al., 2016) according to the number of highly conserved DNA-binding domain repeats they contain. At present, most relevant studies focus on the R2R3-MYB category, such as apple *MdMYB23* (An et al., 2018), strawberry *MYB10* (Castillejo et al., 2020) and grape *VvMYB5a* (Deluc et al., 2006), which regulate anthocyanins and flavonols. In this study, the expression of *MYB90a* (Cluster-28756.143139) was associated with the levels of five cyanidins and was highly expressed in LS. Bac-Molenaar et al. (2015) found that *MYB90* was the key TF with phenotypes ranging from green to completely red/purple rosettes in *Arabidopsis thaliana*, and *MYB90* could regulate flower color of *Arabidopsis thaliana* (Stracke et al., 2001; Niu et al., 2016), and it is speculated that *MYB90* may be involved in the color accumulation of LS (Reddish-brown).

The formation of anthocyanins depends on glycosylation, hydroxylation, acylation, and methoxylation to maintain stability, and this process is controlled by some transporters and other proteins (Fu et al., 2021). In this study, key genes involved in the color formation of LS were identified by WGCNA, and 17 genes exhibiting the highest correlation were selected to construct a network diagram. The results showed that these eight anthocyanins were regulated by 2–8 genes. In WGCNA, one *CHY1* gene (Cluster-28756.128315), two *elf-5A* genes (Cluster-28756.207163; Cluster-28756.207871), one *LOC107827976* gene (Cluster-28756.222137), two *guaA* genes (Cluster-28756.216102; Cluster-28756.177157), one *LOS1* gene (Cluster-28756.206848), two *UTP7* genes (Cluster-28756.225447; Cluster-28756.225995), one *EMB2768* gene (Cluster-28756.145529), one *L195_g025361* (Cluster-28756.193371), and one *TOPP6* gene (Cluster-28756.226581) were shown to target one, one, four, two, one, three, one, one, one, four, two, and three anthocyanins, respectively. These results indicate that these 17 genes might play important roles in color formation in LS, but the specific functions need to be further studied.

## Conclusion

In summary, we comprehensively compared the transcriptomes and metabolite profiles of two colors of *C. tinctoria* flowers (LS and JS) at different developmental stages. The flavonoid metabolites (anthocyanins) and DEGs of *C. tinctoria* were revealed, and a few candidate genes related to the main DAFs/DAAs were identified. Our results will provide new information about the synthesis of secondary metabolites, the regulation of flower color of *C. tinctoria*, and the functional identification of key genes of interest.

## Data Availability Statement

The datasets presented in this study can be found in online repositories. The names of the repository/repositories and accession number(s) can be found below: https://www.ncbi.nlm.nih.gov/, bioproject/PRJNA698724.

## Author Contributions

HJ conceived and designed the experiments, performed the experiments, analyzed the data, prepared figures and tables, authored or reviewed drafts of the manuscript, and approved the final draft. ZL performed the experiments, analyzed the data, and approved the final draft. XJ collected the samples and approved the final draft. YQ conceived and designed the experiments, authored or reviewed drafts of the manuscript, and approved the final draft. All authors contributed to the article and approved the submitted version.

## Conflict of Interest

The authors declare that the research was conducted in the absence of any commercial or financial relationships that could be construed as a potential conflict of interest.

## Publisher’s Note

All claims expressed in this article are solely those of the authors and do not necessarily represent those of their affiliated organizations, or those of the publisher, the editors and the reviewers. Any product that may be evaluated in this article, or claim that may be made by its manufacturer, is not guaranteed or endorsed by the publisher.

## Footnotes

1
http://www.anpel.com.cn/

2
http://www.shimadzu.com.cn/

3
http://www.appliedbiosystems.com.cn/

4
http://trinityrnaseq.sourceforge.net/

5
https://code.google.com/p/corset-project/

6
http://www.ncbi.nlm.nih.gov

7
http://xfam.org/

8
http://www.expasy.ch/sprot

9
http://www.genome.jp/kegg

10
http://www.geneontology.org

11
http://www.ncbi.nlm.nih.gov/COG/

12
https://www.ncbi.nlm.nih.gov/bioproject/PRJNA698724

13
http://bowtie-bio.sourceforge.net
