# Supplementary material for: Comparison of Metabolome and Transcriptome of Flavonoid Biosynthesis in Two Colors of Coreopsis tinctoria Nutt
Source: Front Plant Sci. 2022 Mar 9;13:810422. doi: 10.3389/fpls.2022.810422 (PMC8959828; doi:10.3389/fpls.2022.810422)
Supplement: Supplementary file 1 [file Image_1.pdf]

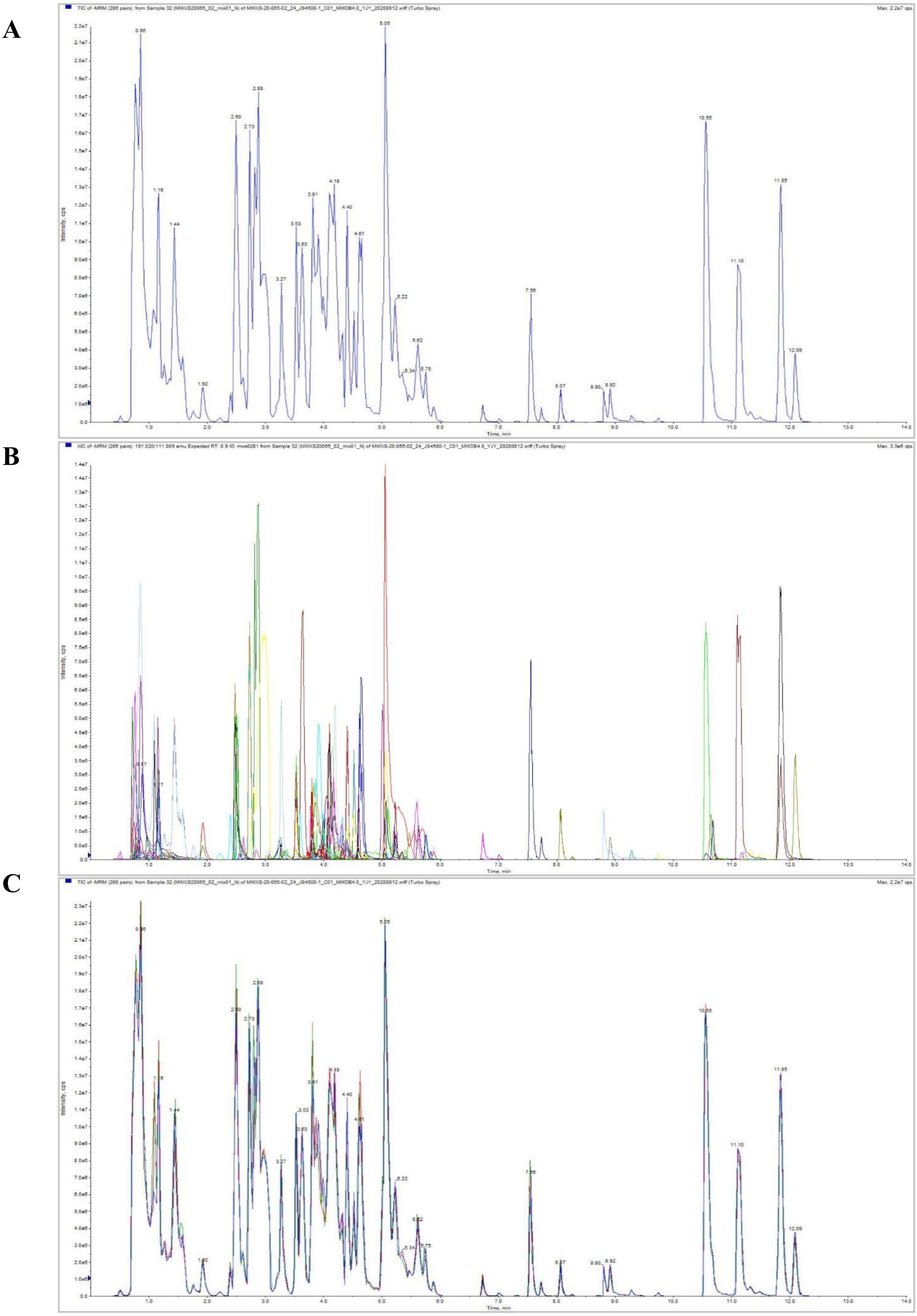

Supplemental Figure 1 | Metabolic spectrum analysis. (**A**) Analysis of total ion flow diagram of mixed samples by mass spectrometry. (**B**) Multi-peak map of MRM metabolite detection. Each different color of the mass spectrum peak represents a metabolite detected. (**C**) TIC overlap map of QC samples detected by mass spectrometry. Abscissa is the retention time of metabolite detection (Retentiontime, Rt), ordinate is the ion current intensity of ion detection (unit of intensity is cps, countpersecond).

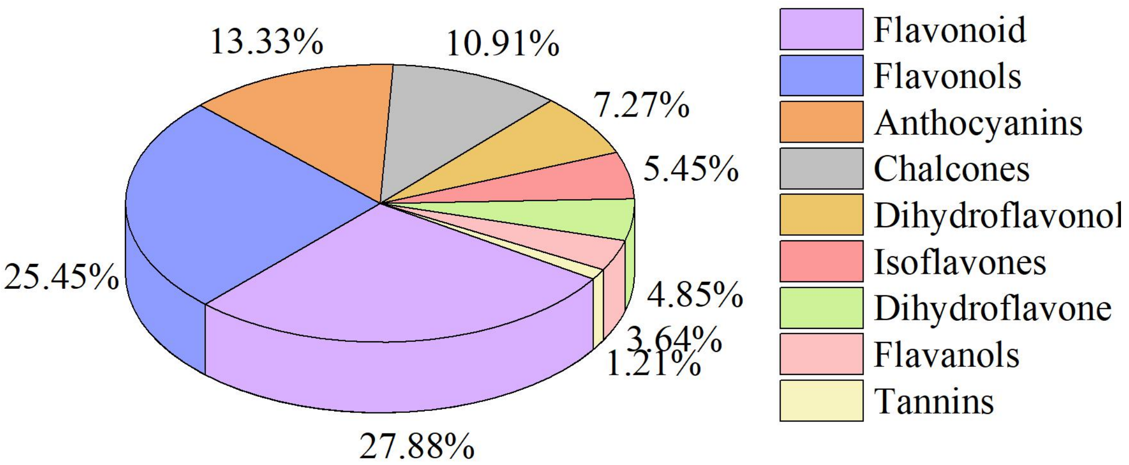

Supplemental Figure 2 | Identification of 165 types of flavonoids in LS and JS.

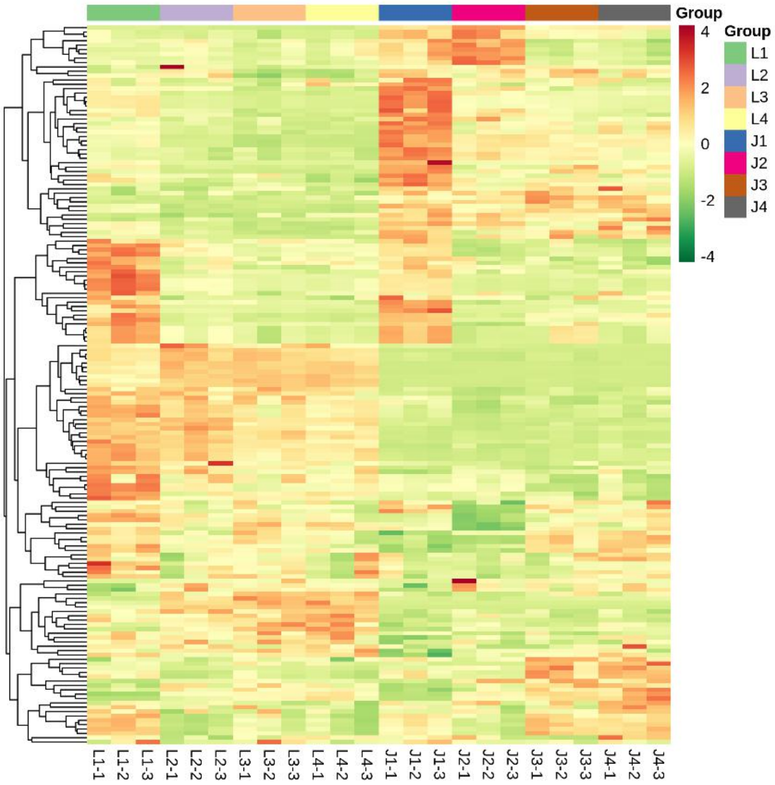

Supplemental Figure 3 | Hierarchical clustering thermographic analysis (HCA) of relative content of flavonoids in LS and JS. The darker the red, the higher the relative content of flavonoid metabolites. The darker the green, the higher the relative content of flavonoid metabolites.

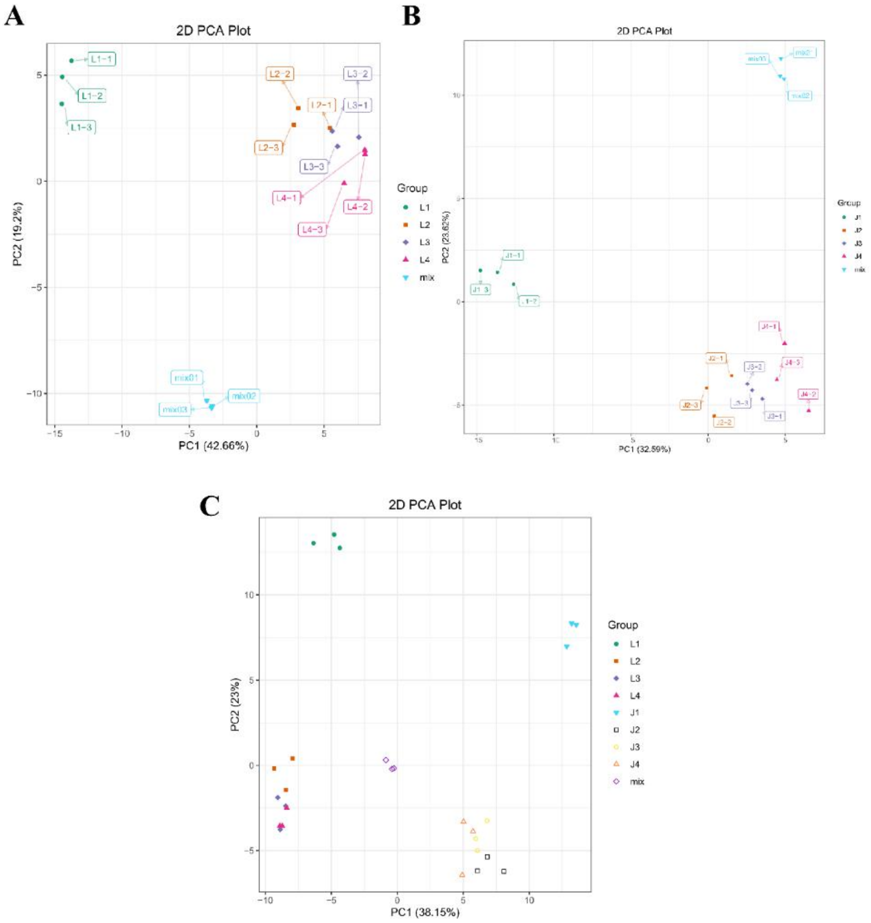

Supplemental Figure 4 | Principal component analysis of samples. (**A**) Principal component analysis of LS samples. (**B**) Principal component analysis of JS samples. (**C**) Principal component analysis of LS and JS samples.

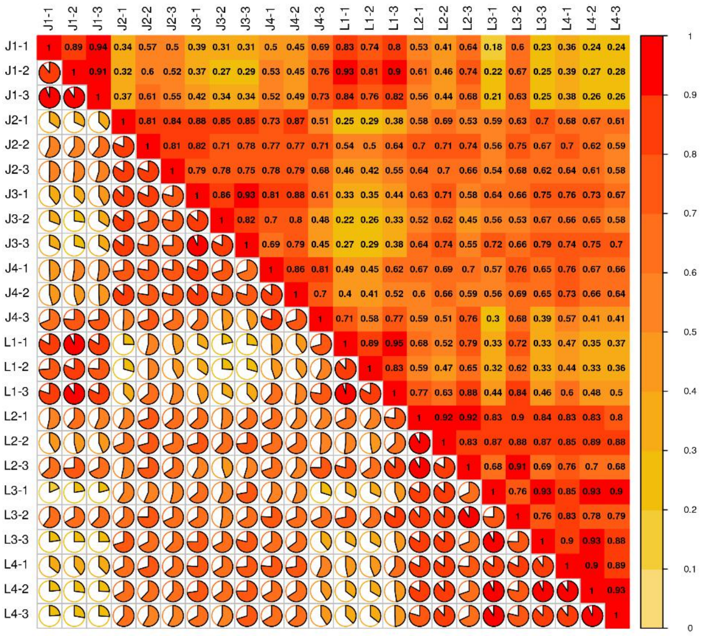

Supplemental Figure 5 | Correlation heat map between transcriptome samples

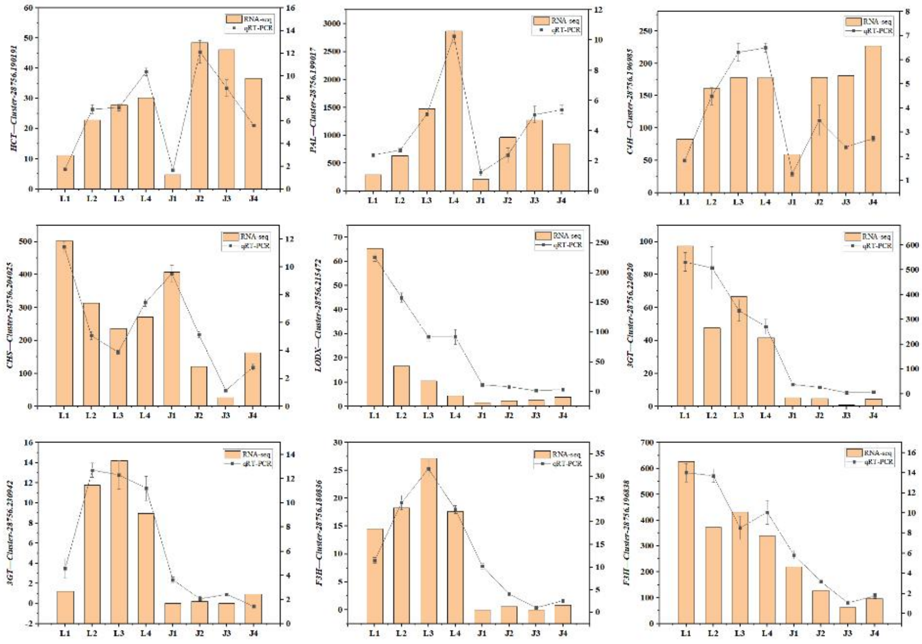

Supplemental Figure 6 | RNA-seq results by qRT-PCR

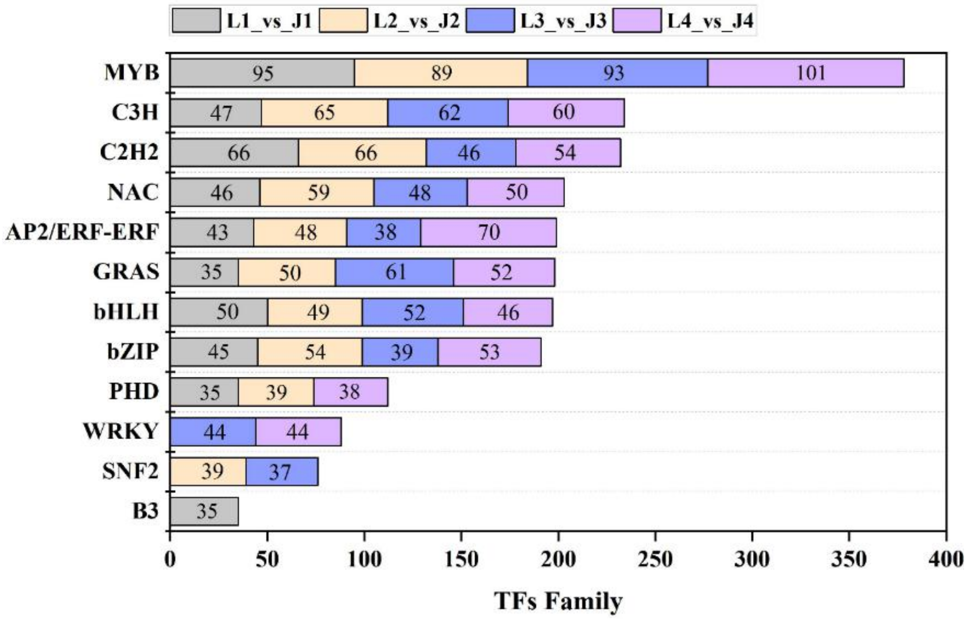

Supplemental Figure 7 | The statistics of the top 10 transcription factors (TFs) with the largest number among LS vs. JS groups.

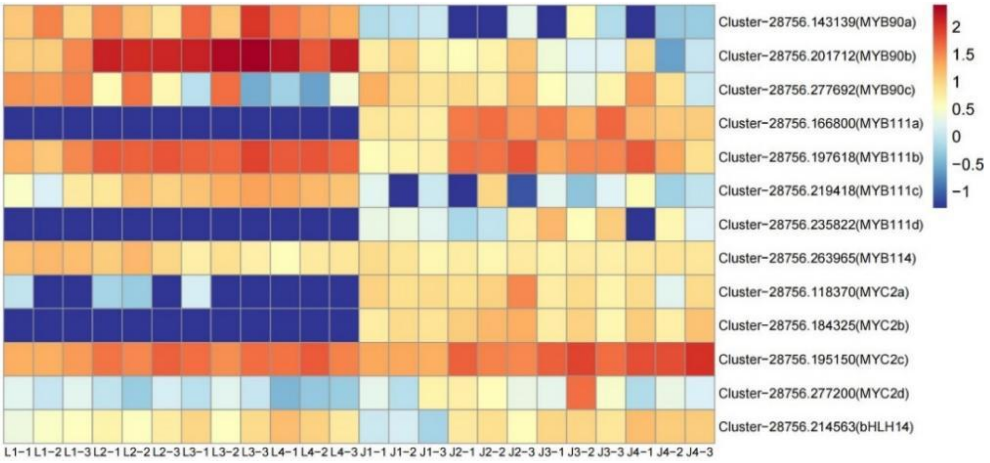

Supplemental Figure 8 | Screening of MYB and bHLH transcription factors (TFs) related to flavonoids synthesis

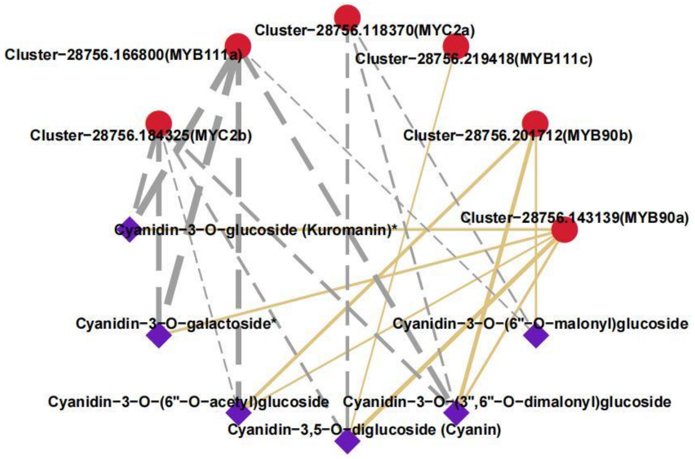

Supplemental Figure 9 | Network diagram of correlation between transcription factors (TFs) and DAAs. The real and virtual lines represent positive and negative correlation, the thickness of lines indicates the strength of correlation; Red for TFs. Purple for DAAs.

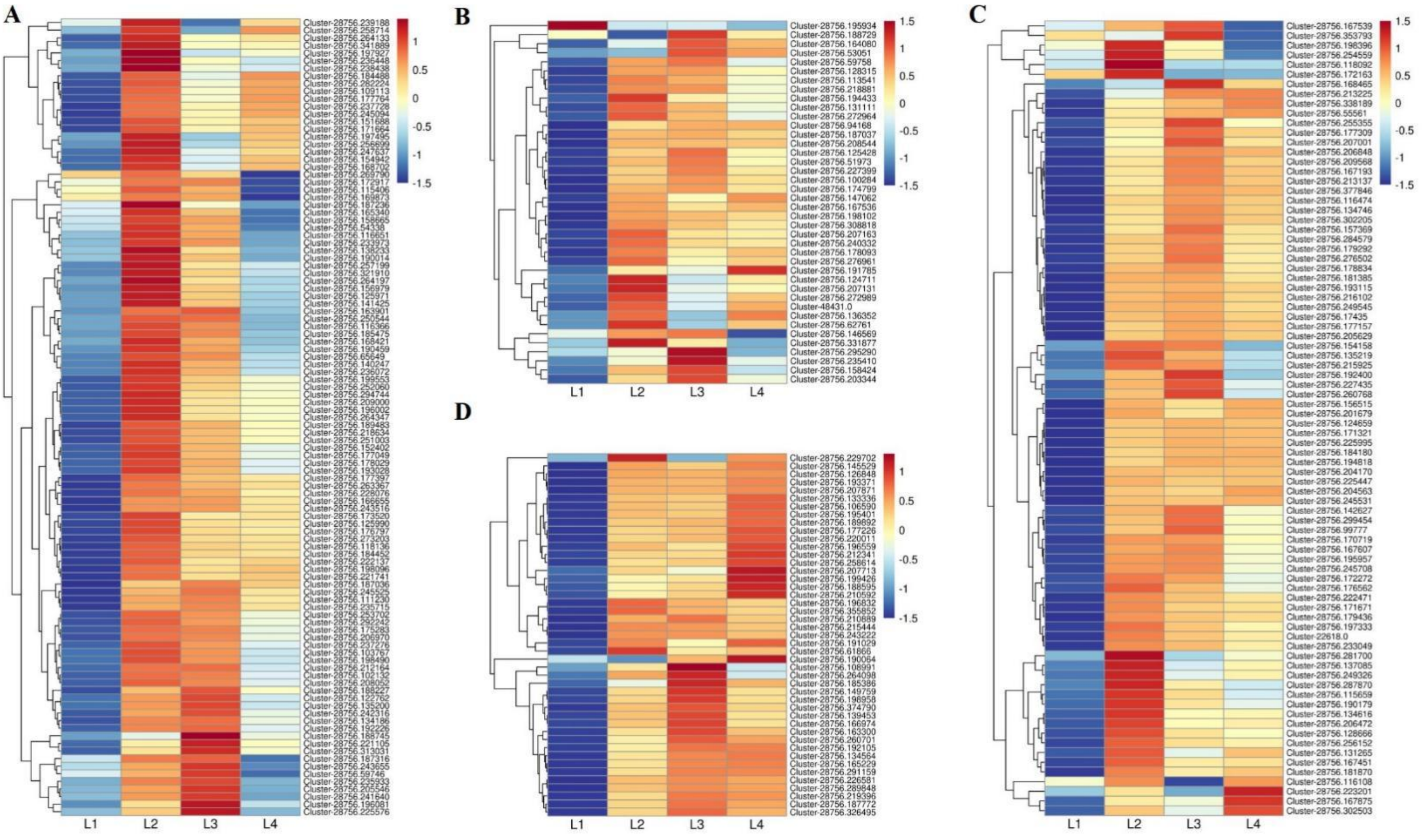

Supplemental Figure 10 | The expression level (FPKM value) of DEGs in four modules. (**A**) 105 DEGs of MEgreen module; (**B**) 40 DEGs of MEdarkgreen module; (**C**) 82 DEGs of MEmagenta module; (**D**) 45 DEGs of MEroyablue module.
