# Supplementary material for: Comparison of Metabolome and Transcriptome of Flavonoid Biosynthesis in Two Colors of Coreopsis tinctoria Nutt
Source: Front Plant Sci. 2022 Mar 9;13:810422. doi: 10.3389/fpls.2022.810422 (PMC8959828; doi:10.3389/fpls.2022.810422)
Supplement: Supplementary file 2 [file Table_1.pdf]

Supplemental Table 1 | The composition of Hoagland solution

Material	Chemical formula	Dosage (mg/L)
Calcium nitrate tetrahydrate	Ca(NO3)2·4H2O	945
Potassium nitrate	KNO_3	607
Ammonium phosphate	$(NH4)_2HPO_4$	115
Magnesium sulfate heptahydrate	$MgSO_4·7H_2O$	493
Ethylenediaminetetraacetic acid disodium salt	EDTA-Na	20-40
Orthoboric acid	H_3BO_3	2.86
Manganese sulphate	$MnSO_4 · 4H_2O$	2.13
Zincsulfate	$ZnSO_4$ · 7H2O	0.22
Copper sulfate	$CuSO_4 · 5H_2O$	0.08
Ammonium molybdate	$(NH4)_2MoO_4$	0.02

Supplemental Table 2 | List of primers used for qRT-PCR

Gene name	Description	Primer Sequence (5'-3')
GAPDH		F: ACAAGCTTCCCACCTTCTCA<br>R: TGGAAGGTTGGAGGGGTTAC
Cluster-28756.190191	*HCT* / shikimate O-hydroxycinnamoyltransferase	F: AGGATCGTGGTGGATGGTTT<br>R: TAGCTATCCCGCCGTTCATT
Cluster-28756.199017	*PAL* / phenylalanine ammonia	F: CCGCATTAAGGATTTCGCCA<br>R: ACAACAACATAACCCCGTGC
Cluster-28756.196985	*C4H* / cinnamate 4-hydroxylase	F: GACGACACCACGACAAGATTG<br>R: CTCTAAGCTACGTGGTAAGAA
Cluster-28756.204025	*CHS* / chalcone synthase	F: GGAAGGTGCGATTGATGGTC<br>R: AACGCTTGAACCAATGCCTT
Cluster-28756.180836	*F3H* / flavanone 3-hydroxylase	F: GGGGAAGGAATCTAGCCGTT<br>R: GGTAAGCTGGAGGTGAGTGT
Cluster-28756.196838		F: GCTAGTTTCTTGAGCCGAGC<br>R: CCGCACCAGAAGCAATAGTG
Cluster-28756.215472	*LDOX* / leucoanthocyanidin dioxygenase	F: TTCTCTTCCACCGGCTGATT<br>R: TGGGGAGTAATGCACCTTGT
Cluster-28756.230942	*3GT* / anthocyanidin 3-O-glucosyltransferase	F: AAAACGCCACCCAAGGAATC<br>R: GGCGGAGAAGGATATTGGGA
Cluster-28756.220920		F: GATGCAGTTGGGGTGTTTGT<br>R: TCTCACCCCGATTTCCCAAA

Supplemental Table 3｜ Information of 165 flavonoid compounds identified.

Index	Q1 (Da)	Q3 (Da)	Ionization model	Formula	Compounds	Class
mws0040	2.55E+02	1.53E+02	[M+H]+	C15H10O4	Chrysin	Flavonoid
Lmmp007480	2.57E+02	1.37E+02	[M+H]+	C15H12O4	2,4,4'-trihydroxychalcone	Flavonoid
pmp000571	2.71E+02	1.53E+02	[M+H]+	C15H10O5	Apigenin	Flavonoid
pmp000344	2.71E+02	2.43E+02	[M+H]+	C15H10O5	3',4',7-Trihydroxyflavone	Flavonoid
Hmbp002730	2.87E+02	1.53E+02	[M+H]+	C15H10O6	Isoscutellarein	Flavonoid
pme0088	2.85E+02	1.51E+02	[M-H]-	C15H10O6	Luteolin (5,7,3',4'-Tetrahydroxyflavone)	Flavonoid
pmp000001	3.01E+02	2.86E+02	[M+H]+	C16H12O6	Hispidulin (5,7,4'-Trihydroxy-6-methoxyflavone)	Flavonoid
mws0058	2.99E+02	2.84E+02	[M-H]-	C16H12O6	Diosmetin (5,7,3'-Trihydroxy-4'-methoxyflavone)	Flavonoid
Lmjp004941	3.01E+02	2.86E+02	[M+H]+	C16H12O6	Rhamnocitrin (7-Methylkaempferol)	Flavonoid
Zmhn003257	2.99E+02	2.84E+02	[M-H]-	C16H12O6	5,7,2'-Trhiyroxy-8-methoxyflavone	Flavonoid
pmp000003	3.17E+02	3.02E+02	[M+H]+	C16H12O7	Nepetin (5,7,3',4'-Tetrahydroxy-6-methoxyflavone)	Flavonoid
Lmjp007790	3.29E+02	3.14E+02	[M+H]+	C18H16O6	5-Hydroxy-3,7,4'-trimethoxyflavone	Flavonoid
Lmjp004404	3.33E+02	3.18E+02	[M+H]+	C16H12O8	Quercetagetin-3-Methyl Ether	Flavonoid
Lmyp005841	4.17E+02	2.55E+02	[M+H]+	C21H20O9	Chrysin-7-O-glucoside	Flavonoid
Lmyp004617	4.19E+02	2.57E+02	[M+H]+	C21H22O9	Pinocembrin-7-O-glucoside (Pinocembroside)	Flavonoid
Zmhp002776	4.31E+02	2.55E+02	[M+H]+	C21H18O10	Chrysin-7-O-Glucuronide	Flavonoid
mws0072	4.33E+02	2.71E+02	[M+H]+	C21H20O10	Apigenin-5-O-glucoside	Flavonoid
Lmlp005572	4.33E+02	2.71E+02	[M+H]+	C21H20O10	Galangin-7-O-glucoside	Flavonoid
HJN087	4.33E+02	2.71E+02	[M-H]-	C21H22O10	Naringenin-4'-O-glucoside	Flavonoid
HJN090	4.33E+02	2.71E+02	[M-H]-	C21H22O10	Butin-7-O-glucoside	Flavonoid
mws0052	4.47E+02	2.71E+02	[M+H]+	C21H18O11	Baicalin	Flavonoid
pme2459	4.49E+02	2.87E+02	[M+H]+	C21H20O11	Luteolin-7-O-glucoside (Cynaroside)	Flavonoid
Xmyp005654	4.49E+02	2.87E+02	[M+H]+	C21H20O11	Kaempferol-4'-O-glucoside*	Flavonoid
pmp000012	4.63E+02	2.87E+02	[M+H]+	C21H18O12	Scutellarein-7-O-glucuronide (Scutellarin)	Flavonoid
pmn001702	4.61E+02	2.85E+02	[M-H]-	C21H18O12	Tetahydroxyflavone-7-O-glucuronide	Flavonoid
mws4167	4.63E+02	2.87E+02	[M+H]+	C21H18O12	Luteolin-7-O-glucuronide	Flavonoid
pmp000579	4.63E+02	3.01E+02	[M+H]+	C22H22O11	Diosmetin-7-O-galactoside	Flavonoid
Lmjp003655	4.63E+02	3.01E+02	[M+H]+	C22H22O11	6-C-MethylKaempferol-3-glucoside	Flavonoid
pmb2999	4.61E+02	2.99E+02	[M-H]-	C22H22O11	Chrysoeriol-5-O-glucoside	Flavonoid
Lmzp002365	4.65E+02	3.03E+02	[M+H]+	C22H24O11	Hesperetin-7-O-glucoside	Flavonoid
Xmsn002700	4.65E+02	3.03E+02	[M-H]-	C21H22O12	Taxifolin-3'-O-glucoside	Flavonoid
pmp000583	4.77E+02	3.01E+02	[M+H]+	C22H20O12	Diosmetin-7-O-glucuronide	Flavonoid
Lmfp003403	4.81E+02	3.19E+02	[M+H]+	C21H20O13	Quercetagetin-7-O-glucoside	Flavonoid
HJAP005	4.95E+02	3.33E+02	[M+H]+	C22H22O13	Laricitrin-3-O-glucoside	Flavonoid
Lmtn002997	4.93E+02	3.31E+02	[M-H]-	C22H22O13	Mearnsetin-3-O-glucoside	Flavonoid
Lmjp003231	4.95E+02	3.33E+02	[M+H]+	C22H22O13	Patuletin-3-O-glucoside	Flavonoid
HJAP135	5.05E+02	2.57E+02	[M+H]+	C24H24O12	Pinocembrin-7-O-(6"-O-malonyl)glucoside	Flavonoid
pmb3000	5.03E+02	3.41E+02	[M-H]-	C24H24O12	Chrysoeriol-7-O-(6"-acetyl)glucoside	Flavonoid
Hmcp002187	5.09E+02	3.47E+02	[M+H]+	C23H24O13	Limocitrin-3-O-galactoside	Flavonoid
Hmcp001636	5.09E+02	3.47E+02	[M+H]+	C23H24O13	Limocitrin-7-O-glucoside	Flavonoid
HJAP006	5.09E+02	3.47E+02	[M+H]+	C23H24O13	Syringetin-7-O-glucoside	Flavonoid
pmp000585	5.19E+02	4.33E+02	[M+H]+	C24H22O13	Apigenin-7-O-(6"-malonyl)glucoside	Flavonoid
pmp000587	5.35E+02	4.49E+02	[M+H]+	C24H22O14	Luteolin-7-O-(6"-malonyl)glucoside	Flavonoid
pmp000588	5.49E+02	4.63E+02	[M+H]+	C25H24O14	Diosmetin-7-O-(6"-malonyl)glucoside	Flavonoid
pmb0608	5.49E+02	3.01E+02	[M+H]+	C25H24O14	Chrysoeriol-7-O-(6"-malonyl)glucoside	Flavonoid
Lmmp003487	6.39E+02	2.87E+02	[M+H]+	C27H26O18	Luteolin-7-O-glucuronide-(2→1)-glucuronide	Flavonoid

pme3514	3.01E+02	1.51E+02	[M-H]-	C15H10O7	Morin*	Flavonols
pme2954	3.03E+02	1.37E+02	[M+H]+	C15H10O7	Quercetin*	Flavonols
Lmmn004912	3.15E+02	2.71E+02	[M-H]-	C16H12O7	Quercetin-3-O-methyl ether	Flavonols
mws0066	3.15E+02	1.51E+02	[M-H]-	C16H12O7	Isorhamnetin	Flavonols
mws0988	3.15E+02	1.65E+02	[M-H]-	C16H12O7	Rhamnetin (7-O-Methxyl Quercetin)	Flavonols
Lmpn007255	3.31E+02	3.16E+02	[M-H]-	C16H12O8	Patuletin (Quercetagetin-6-methyl ether)	Flavonols
mws2186	4.35E+02	3.03E+02	[M+H]+	C20H18O11	Avicularin	Flavonols
Lmdp003509	4.35E+02	3.03E+02	[M+H]+	C20H18O11	Quercetin-3-O-xyloside (Reynoutrin)*	Flavonols
Lmfp004055	4.35E+02	3.03E+02	[M+H]+	C20H18O11	Morin-3-O-xyloside*	Flavonols
mws0913	4.47E+02	2.85E+02	[M-H]-	C21H20O11	Kaempferol-3-O-galactoside (Trifolin)*	Flavonols
Lmzn001894	4.61E+02	2.85E+02	[M-H]-	C21H18O12	Kaempferol-3-O-glucuronide	Flavonols
Lmdp003286	4.65E+02	3.03E+02	[M+H]+	C21H20O12	Isohyperoside	Flavonols
mws1329	4.63E+02	3.01E+02	[M-H]-	C21H20O12	Quercetin-7-O-glucoside	Flavonols
pmp001309	4.65E+02	3.03E+02	[M+H]+	C21H20O12	6-Hydroxykaempferol-7-O-glucoside	Flavonols
mws0856	4.63E+02	3.01E+02	[M-H]-	C21H20O12	Spiraeoside	Flavonols
mws0091	4.63E+02	3.00E+02	[M-H]-	C21H20O12	Quercetin-3-O-glucoside (Isoquercitrin)	Flavonols
mws0061	4.63E+02	3.00E+02	[M-H]-	C21H20O12	Quercetin-3-O-galactoside (Hyperin)	Flavonols
Lmjp002906	4.79E+02	3.17E+02	[M+H]+	C22H22O12	Rhamnetin-3-O-Glucoside*	Flavonols
Lmyp004444	4.79E+02	3.17E+02	[M+H]+	C22H22O12	Tricin-4'-methylether-3'-O-glucoside	Flavonols
Hmcp002207	4.79E+02	3.17E+02	[M+H]+	C22H22O12	Isorhamnetin-7-O-glucoside (Brassicin)*	Flavonols
Lmcp003788	4.79E+02	3.17E+02	[M+H]+	C22H22O12	Isotamarixin	Flavonols
Lmpp003465	4.81E+02	3.19E+02	[M-H]-	C21H20O13	Myricetin-3-O-glucoside	Flavonols
Lmmp001947	4.81E+02	3.19E+02	[M+H]+	C21H20O13	Gossypetin-3-O-glucoside	Flavonols
P3564	4.81E+02	3.19E+02	[M+H]+	C21H20O13	6-Hydroxyquercetin-O-glucoside	Flavonols
Lmmp003271	4.81E+02	3.19E+02	[M+H]+	C21H20O13	Gossypetin-8-O-glucoside	Flavonols
Hmln002199	5.05E+02	3.00E+02	[M-H]-	C23H22O13	Quercetin-3-O-(6"-acetyl)galactoside	Flavonols
Lmdp004892	5.35E+02	2.87E+02	[M+H]+	C24H22O14	Kaempferol-3-O-(6"-malonyl)galactoside*	Flavonols
Lmmp003817	5.35E+02	2.87E+02	[M+H]+	C24H22O14	Kaempferol-3-O-(6"-malonyl)glucoside*	Flavonols
pmp000589	5.51E+02	3.03E+02	[M+H]+	C24H22O15	Quercetin-7-O-(6"-malonyl)glucoside*	Flavonols
Hmln002189	5.49E+02	5.05E+02	[M-H]-	C24H22O15	Quercetin-3-O-(6"-malonyl)galactoside*	Flavonols
Lmtp004126	5.67E+02	3.19E+02	[M+H]+	C24H22O16	Myricetin-3-O-(6"-malony)glucoside	Flavonols
Lmmp002143	5.67E+02	3.19E+02	[M+H]+	C24H22O16	Gossypetin-3-O-(6"-malonyl)glucoside	Flavonols
Hmbp001825	5.97E+02	3.03E+02	[M+H]+	C26H28O16	Quercetin-3-O-sambubioside	Flavonols
Lmjp002596	5.97E+02	3.03E+02	[M+H]+	C26H28O16	Quercetin-3-O-xylosyl(1→2)glucoside	Flavonols
Lmtp004044	5.97E+02	3.03E+02	[M+H]+	C26H28O16	Quercetin-3-O-apiosyl(1→2)galactoside	Flavonols
Lmtp003677	6.27E+02	3.03E+02	[M+H]+	C27H30O17	Quercetin-3-O-sophoroside (Baimaside)	Flavonols
pmp000596	6.27E+02	4.65E+02	[M+H]+	C27H30O17	Quercetin-3-O-(2"-O-galactosyl)glucoside	Flavonols
pmp001310	6.27E+02	3.03E+02	[M+H]+	C27H30O17	6-Hydroxykaempferol-3,6-O-Diglucoside*	Flavonols
pmp001311	6.27E+02	3.03E+02	[M+H]+	C27H30O17	6-Hydroxykaempferol-7,6-O-Diglucoside*	Flavonols
Hmcp001919	6.83E+02	5.21E+02	[M+H]+	C30H34O18	isorhamnetin-3-O-(6"-acetylglucosyl)(1→3)-glucoside	Flavonols
Lmmp002995	7.13E+02	3.03E+02	[M+H]+	C30H32O20	Quercetin-7-O-(2"-malonyl)glucosyl-5-O-glucoside	Flavonols
pmb0709	7.13E+02	4.65E+02	[M+H]+	C30H32O20	Quercetin-7-O-(6"-malonyl)glucosyl-5-O-glucoside	Flavonols
pme3609	2.87E+02	2.13E+02	[M]+	C15H11O6+	Cyanidin	Anthocyanins
pme0442	3.03E+02	2.29E+02	[M]+	C15H11O7+	Delphinidin	Anthocyanins
Smlp002532	4.19E+02	2.87E+02	[M]+	C20H19O10+	Cyanidin-3-O-arabinoside	Anthocyanins
pme3392	4.33E+02	2.71E+02	[M]+	C21H21O10+	Pelargonidin-3-O-glucoside	Anthocyanins
Smlp001915	4.35E+02	3.03E+02	[M]+	C20H19O11+	Delphinidin-3-O-arabinoside	Anthocyanins
pmb0550	4.49E+02	2.87E+02	[M]+	C21H21O11+	Cyanidin-3-O-glucoside (Kuromanin)*	Anthocyanins

pmf0027	4.49E+02	2.87E+02	[M]+	C21H21O11+	Cyanidin-3-O-galactoside*	Anthocyanins
pmf0203	4.63E+02	3.01E+02	[M]+	C22H23O11+	Peonidin-3-O-glucoside	Anthocyanins
Smlp002918	4.63E+02	3.31E+02	[M]+	C22H23O11+	Malvidin-3-O-arabinoside	Anthocyanins
pmb2962	4.73E+02	2.69E+02	[M-2H]-	C23H23O11+	Pelargonidin-3-O-(6''-O-acetyl)glucoside	Anthocyanins
Lmpp003059	4.91E+02	2.87E+02	[M]+	C23H23O12+	Cyanidin-3-O-(6''-O-acetyl)glucoside	Anthocyanins
pmb0554	5.19E+02	2.71E+02	[M]+	C24H23O13+	Pelargonidin-3-O-(6''-O-malonyl)glucoside	Anthocyanins
pmb0542	5.35E+02	2.87E+02	[M]+	C24H23O14+	Cyanidin-3-O-(6''-O-malonyl)glucoside	Anthocyanins
Smlp003276	5.79E+02	3.31E+02	[M]+	C26H27O15+	Malvidin-3-O-(6''-O-malonyl)glucoside	Anthocyanins
pme1793	5.95E+02	2.71E+02	[M]+	C27H31O15+	Pelargonidin-3,5-O-diglucoside	Anthocyanins
pmb0562	6.05E+02	2.71E+02	[M]+	C27H25O16+	Pelargonidin-3-O-(3'',6''-O-dimalonylglucoside)	Anthocyanins
Lmtp003079	6.09E+02	3.01E+02	[M]+	C28H33O15+	Peonidin-3-O-rutinoside	Anthocyanins
Lmpp003662	6.11E+02	3.03E+02	[M]+	C30H27O14+	Delphinidin-3-O-(6''-O-p-coumaroyl)glucoside	Anthocyanins
pme1777	6.11E+02	2.87E+02	[M]+	C27H31O16+	Cyanidin-3,5-O-diglucoside (Cyanin)	Anthocyanins
pmb0557	6.21E+02	2.87E+02	[M]+	C27H25O17+	Cyanidin-3-O-(3'',6''-O-dimalonyl)glucoside	Anthocyanins
Lmcp005542	6.27E+02	3.03E+02	[M]+	C30H27O15+	Delphinidin-3-O-(6''-O-caffeoyl)glucoside	Anthocyanins
Lmqp001828	8.75E+02	7.13E+02	[M]+	C36H43O25+	Delphinidin-3-O-(2'''-O-malonyl)sophoroside-5-O-glucoside	Anthocyanins
pme3217	2.57E+02	1.37E+02	[M+H]+	C15H12O4	Isoliquiritigenin	Chalcones
mws4060	2.71E+02	1.21E+02	[M+H]+	C16H14O4	Echinatin	Chalcones
pme3440	2.73E+02	1.37E+02	[M+H]+	C15H12O5	Butein	Chalcones
pme2960	2.73E+02	1.53E+02	[M+H]+	C15H12O5	Naringenin chalcone	Chalcones
pme1201	2.73E+02	1.67E+02	[M-H]-	C15H14O5	Phloretin	Chalcones
P5465	2.89E+02	1.63E+02	[M+H]+	C15H12O6	Okanin	Chalcones
Xmgp006913	3.41E+02	1.37E+02	[M+H]+	C20H20O5	2,4,2',4'-tetrahydroxy-3'-prenylchalcone	Chalcones
pme1399	3.55E+02	1.79E+02	[M+H]+	C21H22O5	Xanthohumol	Chalcones
pmp000384	4.19E+02	2.57E+02	[M+H]+	C21H22O9	Isoliquiritin*	Chalcones
P5179	4.35E+02	2.73E+02	[M+H]+	C21H22O10	coreopsin isomer*	Chalcones
P4850	4.35E+02	2.73E+02	[M+H]+	C21H22O10	coreopsin*	Chalcones
Lmlp006175	4.35E+02	2.73E+02	[M+H]+	C21H22O10	Isosalipurposide (Phlorizin Chalcone)	Chalcones
mws2118	4.35E+02	1.67E+02	[M-H]-	C21H24O10	Phloretin-2'-O-glucoside (Phlorizin)	Chalcones
P4195	4.51E+02	2.89E+02	[M+H]+	C21H22O11	marein	Chalcones
Hmpn005101	4.51E+02	2.89E+02	[M-H]-	C21H24O11	Sieboldin	Chalcones
P4571	5.37E+02	2.89E+02	[M+H]+	C24H24O14	Okanin-malonylglucoside	Chalcones
pmp000393	5.51E+02	4.19E+02	[M+H]+	C26H30O13	Isoliquiritin apioside	Chalcones
Lmlp007435	5.81E+02	1.47E+02	[M+H]+	C30H28O12	Isosalipurposide-6''-O-p-coumaric acid	Chalcones
mws0914	2.71E+02	1.51E+02	[M-H]-	C15H12O5	Pinobanksin	Dihydroflavonol
mws1000	2.87E+02	1.49E+02	[M-H]-	C15H12O6	Fustin	Dihydroflavonol
mws1094	2.87E+02	2.59E+02	[M-H]-	C15H12O6	Dihydrokaempferol	Dihydroflavonol
mws0044	3.03E+02	1.25E+02	[M-H]-	C15H12O7	Dihydroquercetin(Taxifolin)	Dihydroflavonol
P4582	3.05E+02	2.31E+02	[M+H]+	C15H12O7	8-HydroxyFustin	Dihydroflavonol
mws1174	3.13E+02	2.53E+02	[M-H]-	C17H14O6	3-O-Acetylpinobanksin	Dihydroflavonol
mws1360	4.33E+02	2.69E+02	[M-H]-	C21H22O10	Engeletin	Dihydroflavonol
mws1361	4.49E+02	1.51E+02	[M-H]-	C21H22O11	Astilbin	Dihydroflavonol
Lmlp005236	4.51E+02	2.89E+02	[M+H]+	C21H22O11	Dihydrokaempferol-3-O-glucoside	Dihydroflavonol
pmb2975	4.77E+02	3.57E+02	[M-H]-	C23H26O11	Hesperetin-3'-O-glucuronide	Dihydroflavonol
pmb1240	5.03E+02	3.11E+02	[M+H]+	C26H30O10	Phellodensin F	Dihydroflavonol
pmp000531	5.19E+02	3.57E+02	[M+H]+	C26H30O11	Phellamurin	Dihydroflavonol
mws0063	2.71E+02	2.15E+02	[M+H]+	C15H10O5	Genistein	Isoflavones
Lmmp004504	2.87E+02	1.53E+02	[M+H]+	C15H10O6	2'-Hydroxygenistein	Isoflavones

Lmgn002843	2.85E+02	1.99E+02	[M-H]-	C15H10O6	2'-Hydroxyisoflavone	Isoflavones
mws0062	2.87E+02	2.41E+02	[M+H]+	C15H10O6	Isoluteolin (Orobol)(5,7,3',4'-tetrahydroxyisoflavone)	Isoflavones
Lmgp004731	4.33E+02	2.71E+02	[M+H]+	C21H20O10	5,7,4'-Trihydroxyisoflavone-7-O-galactoside	Isoflavones
pmp001163	4.63E+02	2.86E+02	[M+H]+	C22H22O11	Tectoridin	Isoflavones
pmp000193	5.03E+02	2.55E+02	[M+H]+	C24H22O12	6''-O-Malonyldaidzin	Isoflavones
pmp000194	5.19E+02	2.71E+02	[M+H]+	C24H22O13	6''-O-Malonylgenistin	Isoflavones
Zmdp004112	5.19E+02	2.71E+02	[M+H]+	C24H22O13	Genistein-7-O-(6''-malonyl)glucoside	Isoflavones
mws0789	2.55E+02	1.51E+02	[M-H]-	C15H12O4	Pinocembrin (Dihydrochrysin)	Dihydroflavone
mws0902	2.55E+02	1.35E+02	[M-H]-	C15H12O4	Liquiritigenin	Dihydroflavone
pme0376	2.71E+02	1.51E+02	[M-H]-	C15H12O5	Naringenin (5,7,4'-Trihydroxyflavanone)	Dihydroflavone
mws0064	2.87E+02	1.35E+02	[M-H]-	C15H12O6	Eriodictyol (5,7,3',4'-Tetrahydroxyflavanone)	Dihydroflavone
mws1033	3.03E+02	1.53E+02	[M+H]+	C16H14O6	Homoeriodictyol	Dihydroflavone
pmp000383	4.19E+02	2.57E+02	[M+H]+	C21H22O9	Liquiritigenin-4'-O-Glucoside (Liquiritin)*	Dihydroflavone
mws1179	4.33E+02	2.71E+02	[M-H]-	C21H22O10	Naringenin-7-O-glucoside (Prunin)	Dihydroflavone
mws0057	4.51E+02	2.89E+02	[M+H]+	C21H22O11	Eriodictyol-7-O-glucoside	Dihydroflavone
mws0054	2.89E+02	2.45E+02	[M-H]-	C15H14O6	Catechin	Flavanols
pmc1990	2.99E+02	2.23E+02	[M-H]-	C17H16O5	4'-Hydroxy-5,7-dimethoxyflavanone	Flavanols
mws0042	3.05E+02	1.25E+02	[M-H]-	C15H14O7	Epigallocatechin	Flavanols
HJN041	4.51E+02	2.89E+02	[M-H]-	C21H24O11	Epicatechin glucoside	Flavanols
pma0791	5.21E+02	2.73E+02	[M+H]+	C24H24O13	Naringenin-7-O-(6''-malonyl)glucoside	Flavanols
Lmmp000897	6.11E+02	2.87E+02	[M+H]+	C30H26O14	Gallocatechin-Gallocatechin	Flavanols
Hmln000659	3.31E+02	1.69E+02	[M-H]-	C13H16O10	3-O-Galloyl-glucose	Tannin
pmn001533	6.45E+02	1.69E+02	[M-H]-	C26H30O19	2-O-Di-gallic acyl-Glucoside-Glucoside	Tannin

Supplemental Table 4 | Flavonoids with significant differences of LS and JS. In the figure, the positive number indicates the multiple of up-regulation change and the negative number indicates the multiple of down-regulation change, all of which are expressed by Log_2FC value.

Class	Index	Compounds	L1_vs_J1	L2_vs_J2	L3_vs_J3	L4_vs_J4	Regulated
Anthocyanins	pmb0562	Pelargonidin-3-O-(3",6"-O-dimalonylglucoside)	-6.25	-5.39	-5.05	-4.82	Down
	pmb0554	Pelargonidin-3-O-(6"-O-malonyl)glucoside	-5.96	-5.44	-4.97	-4.86	Down
	pme1777	Cyanidin-3,5-O-diglucoside (Cyanin)	-3.45	-5.64	-5.46	-5.49	Down
	pme3392	Pelargonidin-3-O-glucoside	-5.61	-5.03	-4.39	-4.09	Down
	pmb0557	Cyanidin-3-O-(3",6"-O-dimalonyl)glucoside	-3.16	-3.70	-3.68	-3.71	Down
	Lmpp003059	Cyanidin-3-O-(6"-O-acetyl)glucoside	-1.56	-3.55	-3.57	-3.65	Down
	Smlp002532	Cyanidin-3-O-arabinoside	-4.11	-2.85	-2.68	-2.38	Down
	pmb0542	Cyanidin-3-O-(6"-O-malonyl)glucoside	-2.31	-3.11	-3.05	-2.98	Down
Flavonols	Lmjp002906	Rhamnetin-3-O-Glucoside*	-2.95	-2.59	-2.59	-2.40	Down
	Lmyp004444	Tricin-4'-methylether-3'-O-glucoside	-2.88	-2.52	-2.49	-2.42	Down
Dihydroflavonol	mws1094	Dihydrokaempferol	-1.82	-2.60	-1.98	-2.27	Down
Anthocyanins	pmf0027	Cyanidin-3-O-galactoside*	-1.69	-2.85	-2.19	-1.91	Down
	pmb0550	Cyanidin-3-O-glucoside (Kuromanin)*	-1.67	-2.83	-2.19	-1.93	Down
Flavonoid	Hmcp002187	Limocitrin-3-O-galactoside	-2.99	-2.19	-1.72	-1.05	Down
Dihydroflavonol	mws0914	Pinobanksin	-1.96	-1.68	-1.25	-1.06	Down
Dihydroflavone	pme0376	Naringenin (5,7,4'-Trihydroxyflavanone)	-1.93	-1.69	-1.22	-1.04	Down
Chalcones	pme2960	Naringenin chalcone	-1.82	-1.50	-1.20	-1.03	Down
Flavonoid	pmp000571	Apigenin	-1.41	-2.05	/	/	Down
	HJAP135	Pinocembrin-7-O-(6"-O-malonyl)glucoside	/	/	-1.01	-1.25	Down
	mws0052	Baicalin	-1.50	/	/	/	Down
Flavonols	pme3514	Morin*	/	/	/	1.01	Up
Flavonoid	mws0072	Apigenin-5-O-glucoside	/	1.13	/	/	Up
	pmb0608	Chrysoeriol-7-O-(6"-malonyl)glucoside	/	1.15	/	/	Up
Flavonols	Lmmp003271	Gossypetin-8-O-glucoside		1.16	/	/	Up
Flavonoid	HJAP005	Laricitrin-3-O-glucoside	1.03	/	/	1.23	Up
Flavonols	Lmjp002596	Quercetin-3-O-xylosyl(1→2)glucoside	/	/	1.41	1.22	Up
	Hmbp001825	Quercetin-3-O-sambubioside	/	/	1.45	1.31	Up
	Lmmp002143	Gossypetin-3-O-(6"-malonyl)glucoside	/	/	1.17	1.93	Up
Anthocyanins	Lmcp005542	Delphinidin-3-O-(6"-O-caffeoyl)glucoside	/		1.46	1.72	Up
Flavonols	Lmtp004126	Myricetin-3-O-(6"-malony)glucoside	/	/	1.23	2.06	Up
	Hmcp002207	Isorhamnetin-7-O-glucoside (Brassicin)*	1.35	/	/	2.34	Up
	Lmtp004044	Quercetin-3-O-apiosyl(1→2)galactoside	/	1.22	1.45	1.21	Up
	Lmcp003788	Isotamarixin	1.88	2.68	/	/	Up

Supplemental Table 5｜Summary of Illumina transcriptome sequencing

Sample	Raw Reads	Clean Reads	Q20(%)	Q30(%)	GC Content(%)
L1-1	49 226 964	47 294 296	98.09	94.04	43.88
L1-2	46 225 260	44 463 504	98.17	94.38	43.59
L1-3	51 123 260	48 461 036	98.27	94.46	43.95
L2-1	45 3301 60	43 280 360	98.31	94.51	43.94
L2-2	48 904 114	46 758 752	98.25	94.37	43.88
L2-3	47 450 386	45 287 430	98.20	94.27	44.03
L3-1	47 890 404	44 134 010	98.22	94.33	44.05
L3-2	49 805 116	47 721 310	98.20	94.23	43.64
L3-3	48 115 472	46 483 982	98.18	94.21	43.98
L4-1	49 735 484	47 817 484	98.34	94.56	44.16
L4-2	53 410 008	51 523 378	98.20	94.23	44.01
L4-3	48 664 622	46 944 226	98.22	94.32	44.13
J1-1	47 715 920	45 965 250	98.14	94.15	43.62
J1-2	43 150 330	41 593 334	97.40	92.46	43.70
J1-3	45 182 816	43 346 248	98.06	94.13	43.71
J2-1	44 801 704	42 601 146	97.35	92.35	44.24
J2-2	42 768 396	40 703 558	97.09	91.79	44.08
J2-3	47 984 910	45 453 652	97.35	92.36	43.71
J3-1	47 603 568	45 802 276	97.65	93.03	43.98
J3-2	46 543 008	44 781 918	97.75	93.25	43.85
J3-3	45 931 942	44 153 350	97.60	92.90	43.85
J4-1	48 029 354	46 283 322	97.47	92.62	43.84
J4-2	47 653 700	45 678 394	97.49	92.66	44.09
J4-3	462 88 202	44 504 318	97.58	92.85	43.90

Supplemental Table 6 | Statistical table of assembly results

	Distribution	Transcript	Unigene
Length	200~300	78 578	66 036
	300~400	64 289	62 099
	400~500	52 109	51 443
	500~600	42 305	42 099
	600~700	35 006	34 922
	700~800	28 961	28 923
	800~900	24 295	24 282
	900~1000	20 312	20 304
	1000~1100	17 482	17 478
	1100~1200	14 859	14 855
	1200~1300	12 725	12 723
	1300~1400	10 846	10 844
	1400~1500	9 377	9 376
	1500~1600	7 984	7 983
	1600~1700	6 749	6 749
	1700~1800	5 608	5 608
	1800~1900	4 925	4 925
	1900~2000	4 147	4 147
	⩾ 2000	23 017	23 017
Number		463 574	447 813
Mean Length		786	804
N50		1 049	1 061
N90		376	390
Total Bases		364 277 712	360 051 041

Supplemental Table 7 | Summary of functional annotation for assembled unigenes.

Database	Number of Genes	Percentage (%)
NR	264 237	59.01
Trembl	261 115	58.31
GO	218 402	48.77
SwissProt	172 618	38.55
KEGG	170 959	38.18
Pfam	168 542	37.64
KOG	142 997	31.93
Annotated in at least one Database	270 500	60.40
Total Unigenes	447 813	100

Supplemental Table 8 | KEGG pathway statistics of DEGs enrichment

Groups	KEGG annotation pathway	significantly enriched pathways (P value＜0.05)
L1_vs_J1	137	28
L2_vs_J2	137	26
L2_vs_J3	136	26
L4_vs_J4	137	25

Supplemental Table 9丨The 61 DEGs related to flavonoid synthesis

Gene name	Gene ID	Description
HCT shikimate O-hydroxycinnamoyltransferase (19)	Cluster-28756.120208<br>Cluster-28756.243338<br>Cluster-28756.112851<br>Cluster-28756.224573<br>Cluster-28756.112972<br>Cluster-28756.293442<br>Cluster-28756.257821<br>Cluster-28756.295980<br>Cluster-28756.178701<br>Cluster-28756.252528<br>Cluster-28756.290192<br>Cluster-28756.81340<br>Cluster-28756.252527	shikimate O-hydroxycinnamoyltransferase-like (A)
	Cluster-28756.161840<br>Cluster-28756.177047<br>Cluster-28756.245853	spermidine hydroxycinnamoyl transferase (A)
	Cluster-28756.158007<br>Cluster-28756.223381	shikimate O-hydroxycinnamoyltransferase-like isoform X1 (A)
	Cluster-28756.182389	vinorine synthase-like (A)
PGT1 phlorizin synthase (8)	Cluster-28756.200243<br>Cluster-28756.199749<br>Cluster-28756.189824<br>Cluster-28756.200248<br>Cluster-28756.203018<br>Cluster-28756.230443<br>Cluster-28756.259159<br>Cluster-28756.193173	UDP-glycosyltransferase 88B1-like (A)
F3'H flavonoid 3'-monooxygenase (9)	Cluster-28756.175428<br>Cluster-28756.217818<br>Cluster-28756.243945<br>Cluster-28756.67598<br>Cluster-28756.241415	flavonoid 3'-monooxygenase-like isoform X1 (A)
	Cluster-28756.148853<br>Cluster-28756.143921<br>Cluster-28756.70518<br>Cluster-28756.181207	flavonoid 3'-monooxygenase-like (A)
F3H naringenin 3-dioxygenase (6)	Cluster-28756.182100<br>Cluster-28756.279662<br>Cluster-28756.180836<br>Cluster-28756.165750<br>Cluster-28756.196838<br>Cluster-28756.299649<br>Cluster-28756.230942	naringenin,2-oxoglutarate 3-dioxygenase (A)
CHI chalcone isomerase (5)	Cluster-28756.252485<br>Cluster-28756.200451<br>Cluster-28756.228896	chalcone--flavonone isomerase 3 (A)
	Cluster-28756.216663<br>Cluster-28756.187196	chalcone--flavonone isomerase (A)
DFR bifunctional dihydroflavonol 4-reductase (3)	Cluster-28756.266761<br>Cluster-28756.103266<br>Cluster-28756.342757	dihydroflavonol 4-reductase (A)
C4H trans-cinnamate 4-monooxygenase (2)	Cluster-28756.151433	trans-cinnamate 4-monooxygenase
	Cluster-28756.214451	trans-cinnamate 4-monooxygenase-like (A)
CHS chalcone synthase (2)	Cluster-28756.175423	chalcone synthase-like (A)
	Cluster-28756.204025	chalcone synthase (A)
LDOX leucoanthocyanidin dioxygenase (2)	Cluster-28756.215472<br>Cluster-28756.227869	leucoanthocyanidin dioxygenase-like (A)
3GT anthocyanidin 3-O-glucosyltransferase (2)	Cluster-28756.220920<br>Cluster-28756.230942	kaempferol 3-O-beta-D-galactosyltransferase-like (A)
UF3RT flavonol-3-O-glucoside L-rhamnosyltransferase (1)	Cluster-28756.214949	UDP-glycosyltransferase 79A2-like (A)
CCoAOMT caffeoyl-CoA O-methyltransferase (1)	Cluster-28756.162598	caffeoyl-CoA O-methyltransferase At4g26220 isoform X1 (A)
C3'H coumaroylquinate 3'-monooxygenase (1)	Cluster-28756.86707	cytochrome P450 98A2-like (A)

Supplemental Table 10丨The 17 DEGs related to anthocyanin synthesis of LS

Gene name	Gene ID	Description
CHY1	Cluster-28756.128315	3-hydroxyisobutyryl-CoA hydrolase-like protein 1, mitochondrial (A)
EMB2768	Cluster-28756.145529	tyrosine--tRNA ligase, chloroplastic/mitochondrial (A)
GATB	Cluster-28756.138233	glutamyl-tRNA(Gln) amidotransferase subunit B, chloroplastic/mitochondrial (A)
SIGC	Cluster-28756.140247	RNA polymerase sigma factor sigC isoform X1 (A)
MSS116	Cluster-28756.208052	DEAD-box ATP-dependent RNA helicase 31-like (A)
ELF5A	Cluster-28756.207163	eukaryotic translation initiation factor 5A-like (A)
	Cluster-28756.207871	eukaryotic translation initiation factor 5-like (A)
LOS1	Cluster-28756.206848	elongation factor 2 (A)
MRD1	Cluster-28756.213137	multiple RNA-binding domain-containing protein 1 (A)
UTP7	Cluster-28756.225447<br>Cluster-28756.225995	probable U3 small nucleolar RNA-associated protein 7 (A)
L195_g025361	Cluster-28756.193371	ethylene-responsive transcription factor RAP2-12-like (A)
TOPP6	Cluster-28756.226581	serine/threonine-protein phosphatase 6 regulatory subunit 3-like isoform X1 (A)
EMB2768	Cluster-28756.145529	tyrosine--tRNA ligase, chloroplastic/mitochondrial (A)
GATB	Cluster-28756.138233	glutamyl-tRNA(Gln) amidotransferase subunit B, chloroplastic/mitochondrial (A)
SIGC	Cluster-28756.140247	RNA polymerase sigma factor sigC isoform X1 (A)
MSS116	Cluster-28756.208052	DEAD-box ATP-dependent RNA helicase 31-like (A)
LOC107827976	Cluster-28756.222137	ABC transporter B family member 1-like (A)
CHY1	Cluster-28756.128315	3-hydroxyisobutyryl-CoA hydrolase-like protein 1, mitochondrial (A)
PRR73	Cluster-28756.236072	two-component response regulator-like PRR73 (A)
guaA	Cluster-28756.216102<br>Cluster-28756.177157	uncharacterized protein LOC110864408 (A)
